# Development of Dithioacetal‐Modified Dihydropyrimidone Derivatives with Potential Anti‐Inflammatory Activity for Inflammatory Bowel Disease

**DOI:** 10.1002/cbdv.71729

**Published:** 2026-09-25

**Authors:** Jian‐Wei Jiang, Rui Dong, Qi Cao, Huan Wang, Si‐Yu Li, Guo‐Ping Zhang, Qian‐Yi Wang

**Affiliations:** ^1^ Key Laboratory of Green and Precise Synthentic Chemistry and Applications Ministry of Education Huaibei Normal University Huaibei P. R. China; ^2^ College of Chemistry and Chemical Engineering Huaibei Normal University Huaibei P. R. China

**Keywords:** anti‐inflammatory, dihydropyrimidinone, dithioacetal, MAPK/NF‐кB, IBD

## Abstract

Inflammatory bowel disease (IBD) is a chronic autoimmune disorder, and the development of novel anti‐inflammatory small molecules is crucial for advancing therapeutic options. In this study, we designed and synthesized a series of novel dihydropyrimidinone derivatives containing dithioacetal groups. Through in vitro safety assessments and anti‐inflammatory screening, we identified several compounds with promising biological activities. Notably, compound **D3** exhibited exceptional anti‐inflammatory effects, demonstrating an IC_50_ of 1.2 µM in inhibiting NO secretion. Mechanistic investigations revealed that **D3** inhibits the activation of the MAPK/NF‐κB signaling pathway, thereby reducing the expression of LPS induced pro‐inflammatory proteins and the secretion of NO. In vivo, compound **D3** effectively ameliorated DSS‐induced acute colitis in mice, as evidenced by reductions in body weight loss and colonic adhesion. In conclusion, we have developed a series of promising anti‐inflammatory small molecules that may offer significant therapeutic potential for IBD and contribute to the development of new anti‐inflammatory drugs.

## Graphical abstract

This study designed and synthesized dithioacetal‐modified dihydropyrimidinone derivatives (30). Compound D3 showed optimal anti‐inflammatory activity, inhibiting the MAPK/NF‐κB pathway, reducing NO secretion (IC_50_ = 1.2 µM), and ameliorating DSS‐induced acute colitis in mice, offering a new candidate for inflammatory bowel disease therapy.

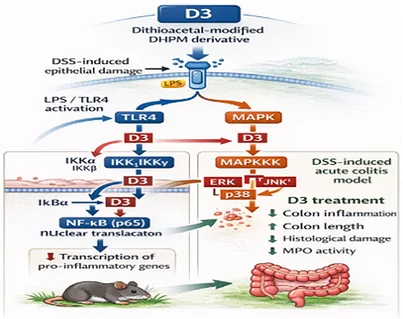

**Abbreviations**
^1^H NMR: proton nuclear magnetic resonance
^13^C NMR: Carbon nuclear magnetic resonance
HR‐MS: High‐resolution electron impact mass spectra
DMF: N, N‐dimethylformamide
THF: Tetrahydrofuran
MeOH: Methanol
EtOAc: Ethyl acetate
DCM: Dichloromethane
DIPEA: N, N‐Diisopropylethylamine
TEA: Trithylamine
IR: Inhibitory rate
IC_50_
: Half maximal inhibitory concentration.

## 1 Introduction

Inflammatory bowel diseases (IBD), comprising ulcerative colitis (UC) and Crohn's disease (CD), affect up to 0.5% of the general population [1, 2]. Recent studies have suggested that IBD might be connected with the loss of gut microbial immunological tolerance [3]. IBD has become a global disease because of the increasing incidence rate caused by the continuous progress of global industrialization, urbanization, and improving diagnosis [4]. The etiology of IBD is unclear so far, although the disorder exhibits genetic susceptibility, and its occurrence and development have been associated with immune disorders, environmental factors, and mucosal dysfunction [5, 6, 7]. Currently, most of the drugs for IBD used commonly in the clinic have adverse reactions, and only a few drugs present long‐lasting treatment effects [8]. Moreover, issues of drug resistance and disease recurrence are frequent and difficult to resolve. Together, these issues cause difficulties in treating patients with IBD. Therefore, it is imperative to develop novel anti‐inflammatory agents for the prevention and treatment of IBD.

In the treatment of IBD patients, traditional clinical drugs such as aminosalicylates, corticosteroids, and can provide symptom relief to some extent [9, 10]. However, their therapeutic effects are relatively limited, prone to relapse, and prolonged use can lead to side effects. Specifically, corticosteroids may cause adverse clinical effects such as osteoporosis and metabolic disorders, while the use of immunosuppressants may increase the risk of infections and cancer in clinical settings [11, 12, 13, 14]. Overall, current clinical treatment strategies for IBD have significant limitations. Therefore, the development of new skeletal compounds with anti‐inflammatory activity is of great significance for the treatment and drug development of IBD.

**FIGURE 1:**
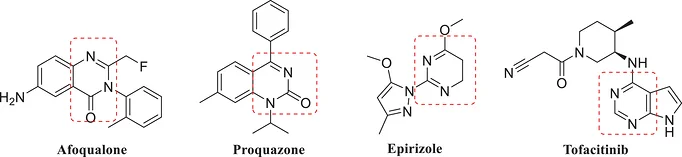
The commercially representative pyrimidine anti‐inflamatory drugs.

Hetero‐cyclic compounds are also present in large variety of drug candidate [15]. The pyrimidine is the most important hetero‐cyclic moiety. Pyrimidine derivatives have various therapeutic applications in medicinal chemistry especially their anti‐inflammatory effects. Several pyrimidine analogs such as afoqualone, proquazone, epirizole, and tofacitinib have already been approved as anti‐inflammatory drugs and are in clinical use (Figure 1) [16, 17, 18, 19].

**FIGURE 2:**
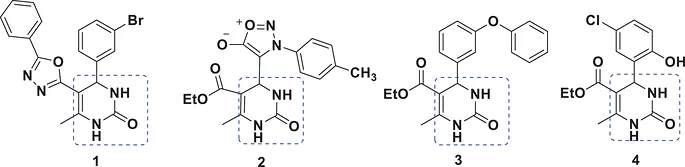
Example of dihydropyrimidinones that exhibit anti‐inflammatory effect.

Research has found that the anti‐inflammatory effects of various synthetic and natural pyrimidines by inhibiting vital inflammatory mediators such as PGE2, nitric oxide (NO), nuclear factor kappa‐light‐chain‐enhancer of activated B cells (NF‐кB), chemokines and cytokines [20]. Dihydropyrimidinones are pyrimidine analogs, the products of the Biginelli reaction are widely used in the pharmaceutical industry as anticancer [21], calcium channel inhibitors [22], anti‐inflammatory [23], antimirobial [24], and antioxidant agents [25]. Especially, it is noteworthy that dihydropyrimidinones (Figure 2) exhibited good anti‐inflammatory role [26]. It has been considered a promising scaffold for the designer of anti‐inflammatory agent. On the other hand, dithioacetal groups have received extensive attention due to their excellent biological activities such as antimicrobial, antibacterial, antileishmanial, antiviral, anticonvulsant, and antidepressant activities [27, 28, 29, 30, 31, 32]. In addition, the introduction of dithioacetal groups into target compounds is implemented to the benefit of their absorption and dissolution. Dihydropyrimidinone derivatives have attracted considerable attention as anti‐inflammatory scaffolds because of their ability to regulate inflammatory mediators and signaling pathways associated with NF‐κB activation. In parallel, dithioacetal‐containing molecules possess a wide spectrum of biological activities and may improve the physicochemical and pharmacokinetic properties of lead compounds. Therefore, based on a molecular hybridization strategy, we envisioned that combining the dihydropyrimidinone pharmacophore with a flexible dithioacetal fragment could generate novel anti‐inflammatory agents with enhanced biological activity.

**FIGURE 3:**
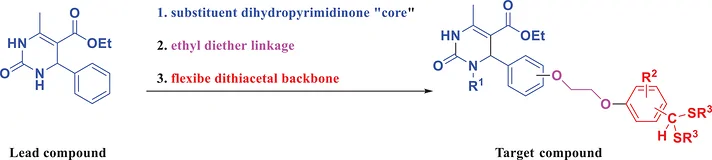
Design strategy of target compound.

Molecular hybridization is a commonly used strategy in drug design, typically achieved by combining different pharmacophoric groups into a new molecular structure to enhance activity and reduce toxicity. In recent years, the team has focused on the research and development of anti‐inflammatory small molecules. Hence, a new series of dihydropymidione derivatives containing a dithioacetal substructure were designed and synthesized (Figure 3). The anti‐inflammation activity of the compounds was evaluated, and preliminary structure‐activity relationship (SAR) and mechanism of action were also discussed. In vitro activity screening results showed that the majority of compounds exhibited good biological activity, with compound D3 showing the strongest inhibitory effect. Further mechanistic studies revealed that compound **D3** effectively inhibited the phosphorylation of key proteins in the MAPK and NF‐κB signaling pathways, suggesting that it may exert its anti‐inflammatory effects by modulating these pathways. Moreover, in vivo experimental results demonstrated that compound **D3** significantly alleviated symptoms in the DSS‐induced acute colitis mouse model. Taken together, these results suggest that this study provides valuable insights for the development of drugs targeting inflammatory bowel disease.

## 2 Results and Discussions

### 2.1 Chemistry

**SCHEME 1:**
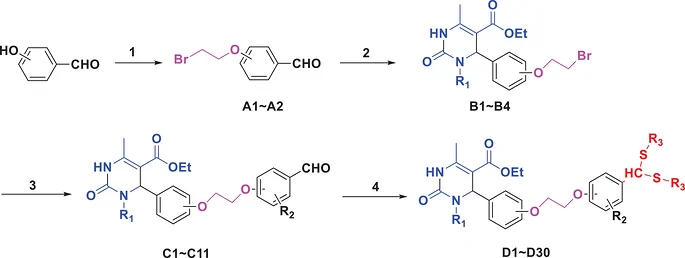
General synthesis of title compounds (**D1**∼**D30**). Reagents and conditions: (1) BrCH_2_CH_2_Br, K_2_CO_3_, CH_3_CN, 80°C, 6 h; (2) CH_3_COCH_2_COOEt, NH_2_CONH_2_ or NH_2_CONHCH_3_, CuCl_2_H_2_O, 90°C, 3 h; (3) Hydroxyaromatic aldehyde, K_2_CO_3_, CH_3_CN, 80°C, 6 h; (4) thiol or thiophenol, ionic liquid [(CH_2_)_3_SO_3_HMIM]HSO_4_, CH_2_Cl_2_, 40°C, 6 h.

A series of novel dihydropymidione derivatives containing a dithioacetal substructure were synthesized and the general pathway was outlined in Scheme 1. The intermediate structure A, which was prepared as previously described was reacted with ethyl acetoacetate and urea (or methylurea), in the presence of copper chloride dihydrate catalyst to give the intermediate B. Then the intermediate C was prepared by the substitution reaction of the intermediate B and substituted hydroxybenzaldehyde. The title compound D was obtained from condensation reaction of the intermediate C and thiol (thiophenol) according to the previous literature. All the target compounds were given spectroscopic analysis, which showed that all compounds were in full accordance with the structures depicted above (Table 1).

**TABLE 1:** The structure of the target compounds.

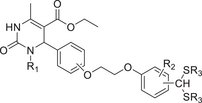					
Compd.	R_1_	R_2_	R_3_	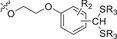	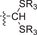
**D1**	H	H	*4‐fluorophenyl*	4‐	4‐
**D2**	H	H	*4‐chlorophenyl*	4‐	4‐
**D3**	H	H	*2‐allyl*	4‐	4‐
**D4**	H	H	*2‐hydroxyethyl*	3‐	4‐
**D5**	H	H	*2‐hydroxyethyl*	4‐	4‐
**D6**	H	H	*2‐hydroxyethyl*	4‐	3‐
**D7**	CH_3_	H	*2‐hydroxyethyl*	4‐	4‐
**D8**	CH_3_	H	*2‐hydroxyethyl*	3‐	4‐
**D9**	CH_3_	H	*4‐fluorophenyl*	4‐	4‐
**D10**	CH_3_	H	*4‐chlorophenyl*	4‐	4‐
**D11**	CH_3_	H	*4‐chlorophenyl*	3‐	4‐
**D12**	H	H	*2‐hydroxyethyl*	3‐	3‐
**D13**	CH_3_	H	*2‐hydroxyethyl*	4‐	3‐
**D14**	H	H	*4‐chlorophenyl*	3‐	3‐
**D15**	CH_3_	H	*4‐methylphenyl*	4‐	4‐
**D16**	CH_3_	H	*4‐methylphenyl*	3‐	4‐
**D17**	CH_3_	H	*4‐methylphenyl*	3‐	3‐
**D18**	H	H	*4‐methylphenyl*	4‐	4‐
**D19**	H	H	*4‐methylphenyl*	4‐	3‐
**D20**	H	H	*4‐methylphenyl*	3‐	3‐
**D21**	H	H	*4‐methylphenyl*	3‐	4‐
**D22**	H	H	*4‐chlorophenyl*	3‐	4‐
**D23**	H	H	*4‐fluorophenyl*	3‐	3‐
**D24**	CH_3_	2‐Cl	*2‐hydroxyethyl*	4‐	4‐
**D25**	CH_3_	2‐Cl	*4‐methylphenyl*	4‐	4‐
**D26**	CH_3_	2‐Cl	*4‐chlorophenyl*	4‐	4‐
**D27**	CH_3_	2‐Cl	*4‐fluorophenyl*	4‐	4‐
**D28**	CH_3_	H	*2‐hydroxyethyl*	3‐	3‐
**D29**	H	2‐Cl	*2‐allyl*	4‐	4‐
**D30**	H	2‐F	*2‐allyl*	4‐	4‐

### 2.2 Compounds Safety Evaluation

It is of great significance to clarify the cytotoxicity of compounds, avoid false positives in detecting their anti‐inflammatory activity, and conduct further research on target compounds. The experimental results showed that compounds did not exhibit any cytotoxicity for RAW264.7 cells with or without LPS stimulation, with an IC_50_ > 50 µM (Table 2). The experimental results showed that the designed and synthesized dihydropyrimidinone derivatives exhibited good cellular safety, which is of great significance for further screening of anti‐inflammatory activity.

**TABLE 2:** Inhibition rate and IC_50_ value of compounds **D1**∼**D30** on NO release and cytotoxicity results.

Compd.	Inhibition rate% (10 µM)	NO IC_50_ (µM)	Cytotoxicity (µM)	
			Without LPS	With LPS
D1	77.93 ± 4.22	3.7	>50	>50
D2	32.34 ± 5.13	>10	>50	>50
D3	98.12 ± 3.16	1.2	>50	>50
D4	57.60 ± 4.16	6.9	>50	>50
D5	48.95 ± 4.03	>10	>50	>50
D6	62.43 ± 4.15	5.3	>50	>50
D7	69.31 ± 5.16	5.3	>50	>50
D8	54.04 ± 4.15	>10	>50	>50
D9	58.83 ± 5.06	8.0	>50	>50
D10	35.39 ± 4.04	>10	>50	>50
D11	23.28 ± 4.19	>10	>50	>50
D12	67.37 ± 4.19	7.5	>50	>50
D13	54.34 ± 5.19	>10	>50	>50
D14	56.14 ± 3.29	>10	>50	>50
D15	26.78 ± 5.06	>10	>50	>50
D16	25.48 ± 4.99	>10	>50	>50
D17	56.15 ± 6.19	7.9	>50	>50
D18	67.34 ± 7.06	5.7	>50	>50
D19	95.00 ± 4.25	1.3	>50	>50
D20	78.72 ± 6.15	5.4	>50	>50
D21	44.40 ± 6.15	>10	>50	>50
D22	53.76 ± 3.16	8.9	>50	>50
D23	68.44 ± 6.06	6.4	>50	>50
D24	85.32 ± 4.15	4.1	>50	>50
D25	33.94 ± 4.71	>10	>50	>50
D26	34.35 ± 7.16	>10	>50	>50
D27	49.17 ± 4.19	>10	>50	>50
D28	74.31 ± 6.05	6.3	>50	>50
D29	70.25 ± 3.05	9.2	>50	>50
D30	94.78 ± 6.32	1.21	>50	>50
Indomethacin	23.56 ± 4.18	>10	>50	>50

### 2.3 Screening of Compounds that Inhibit NO Secretion

Nitric oxide (NO) is a multifunctional signaling molecule involved in numerous physiological processes. While physiological levels of NO exert cytoprotective and regulatory functions, excessive NO production, particularly by inducible nitric oxide synthase (iNOS) under inflammatory conditions has been implicated in the pathogenesis of various inflammatory diseases. Therefore, inhibition of excessive iNOS‐derived NO production has been considered a promising therapeutic strategy for the treatment of inflammatory disorders. To evaluate the anti‐inflammatory effect of dihydropyrimidone derivatives, Griess reagent was used to detect the level of lipopolysaccharide (LPS)‐induced NO release in RAW264.7 cells. As shown in Table 2, Among them, Compounds **D1**, **D3**, **D19**, **D20**, **D24**, **D28,** and **D30** significantly alleviated the increase of LPS‐induced NO release. To better understand the structural features governing anti‐inflammatory activity, a systematic SAR analysis was performed based on the NO inhibition data presented in Table 2. First, the effect of R3 substitution was investigated. Compounds containing aliphatic dithioacetal substituents generally showed superior activity to those bearing aromatic substituents. In particular, allyl‐containing derivatives **D3** and **D30** exhibited the strongest activity with IC_50_ values of 1.2 µM and 1.21 µM, respectively, suggesting that flexible aliphatic groups are beneficial for anti‐inflammatory activity. Second, the substitution pattern of the aromatic linker (R2) significantly affected activity. For compounds sharing the same R3 substituent, para‐substituted analogues tended to display higher activity than their meta‐substituted counterparts. For example, **D5** showed improved activity compared with **D4**, and **D18** was more potent than **D21**. These findings suggest that para‐substitution may provide a more favorable molecular conformation. Third, modification of the dihydropyrimidinone core (R1) influenced biological activity. Comparison of paired analogues indicated that N‐methyl substitution generally decreased activity. This observation implies that the NH group may play a favorable role in maintaining anti‐inflammatory potency. Finally, additional halogen substitution on the aromatic ring did not produce a consistent enhancement in activity. Therefore, excessive aromatic substitution appears to be less favorable than maintaining a relatively simple scaffold combined with a flexible dithioacetal fragment. Taken together, the SAR analysis suggests that compounds possessing an unsubstituted dihydropyrimidinone core, para‐oriented aromatic linker, and flexible aliphatic dithioacetal substituent provide the most promising anti‐inflammatory profile.

### 2.4 Compound D3 Inhibited LPS‐Induced Inflammatory Mediators in RAW264.7 Cell

**FIGURE 4:**
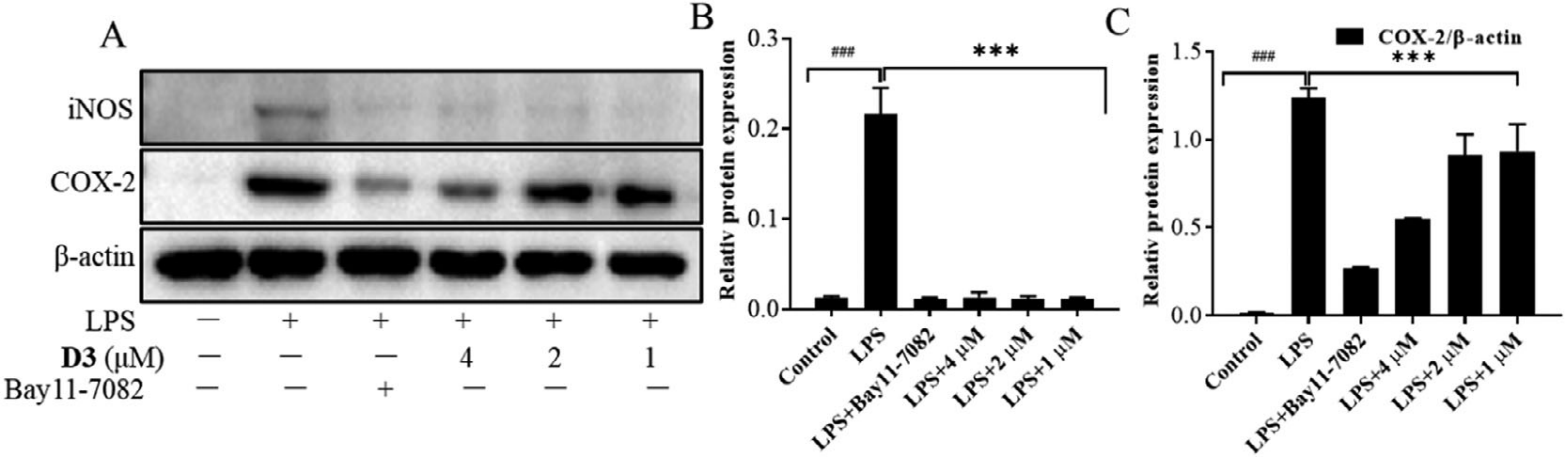
Inhibitory effect of compound **D3** on LPS‐induced protein expression of iNOS and COX‐2. Bay11‐7082 (1 µM) was used as a positive control. All data are presented as mean ± standard deviation (SD), *n* = 3 independent biological replicates. ^###^ *p* < 0.001 vs Control; ***p < 0.001 vs compound **D3** treatment group.

NO, catalyzed by inducible nitric oxide synthase (iNOS) is a hallmark product of inflammatory reactions. High concentrations of NO exhibit bactericidal properties; however, excessive NO release can induce systemic inflammation, playing a pivotal role in inflammatory pathogenesis. Cyclooxygenase‐2 (COX‐2) is an important enzyme involved in regulating inflammatory responses and pain perception, closely associated with disease progression. Therefore, assessing the impact of compounds on iNOS and COX‐2 protein expression is of significant importance. Experimental results (Figure 4) demonstrate that LPS stimulation markedly increases the expression of COX‐2 and iNOS proteins, whereas compound **D3** effectively inhibits the production of iNOS and COX‐2. Furthermore, compound **D3** exhibits a dose‐dependent inhibitory effect on the protein expression of iNOS and COX‐2. These experimental findings indicate that compound **D3** can suppress the expression of the pro‐inflammatory proteins iNOS and COX‐2. Thus, we hypothesize that compound **D3** may influence the upstream signaling pathways of iNOS and COX‐2 proteins.

### 2.5 Compound D3 Inhibited LPS‐Induced NF‐кB Activation

**FIGURE 5:**
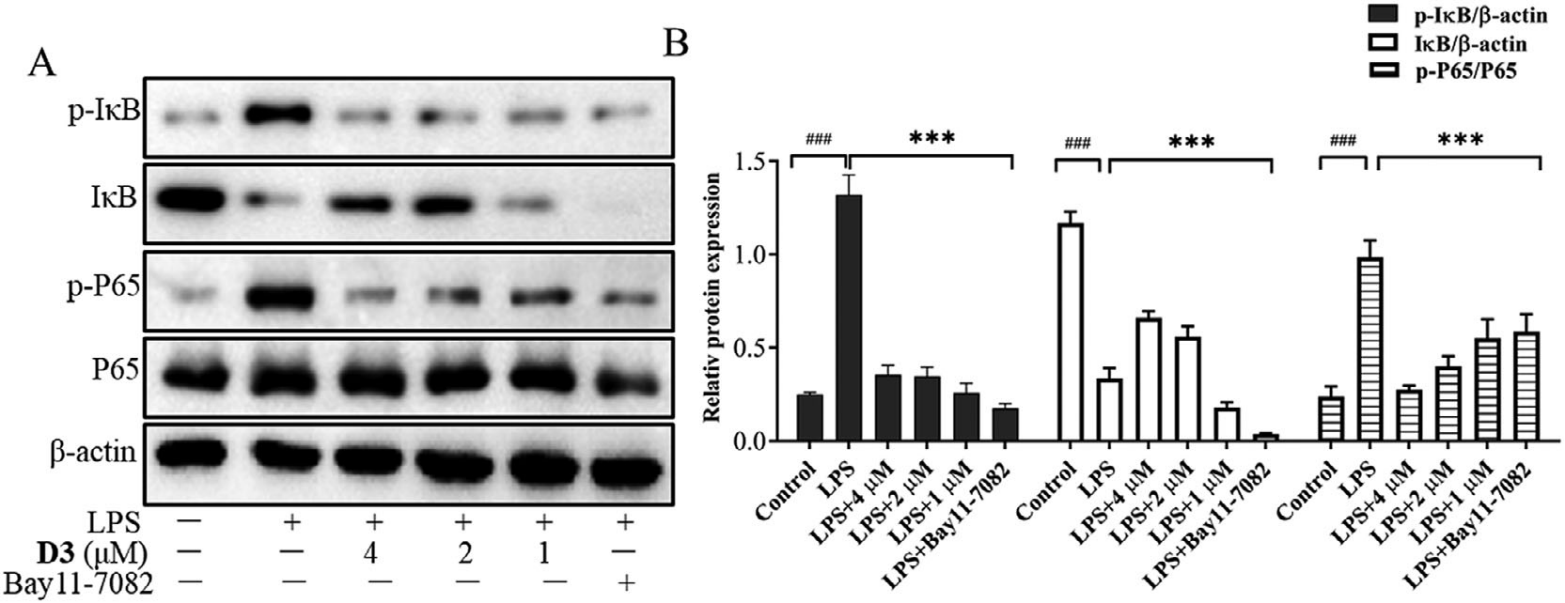
Compound **D3** inhibits LPS‐induced activation of NF‐κB signaling pathway. Bay11‐7082 (1 µM) was used as a positive control. All data are presented as mean ± standard deviation (SD), *n* = 3 independent biological replicates. ^###^ *p* < 0.001 versus Control; ***p < 0.001 vs compound **D3** treatment group.

NF‐κB is a transcription factor protein complex that regulates DNA transcription, cytokine production, and cell survival. Upon cellular stimulation, various signaling pathways are activated, leading to the degradation of IκB and subsequent release of NF‐κB complexes into the nucleus. These complexes bind to specific DNA sites containing NF‐κB, inducing the transcription and expression of specific genes. Activation of these genes by NF‐κB leads to corresponding physiological responses, such as the expression of cytokines, chemokines, iNOS, COX‐2, among others, thereby triggering inflammatory or immune responses in the organism. In order to understand the effect of compound **D3** on NF‐кB signaling LPS‐induced, the relative levels of proteins p‐IкB, IкB, p‐p65, and p65 were examined by western blot. As shown in Figure 5, LPS markedly upreglated the expressions of p‐p65 and p‐IкB compared to control group. However, compound **D3** concentration‐dependently inhibited the expressions of p‐IкB and p‐p65 proteins by LPS‐induced, preliminary indicating that compound **D3** could inhibit the activation of NF‐кB. According to the results, we believe that compound **D3** could regulated the expression of pro‐inflammatory proteins through inhibition of NF‐кB proteins through inhibition of NF‐кB signaling pathways.

### 2.6 Compound D3 Suppresses LPS‐Induced MAPK Activation in RAW264.7 Cells

**FIGURE 6:**
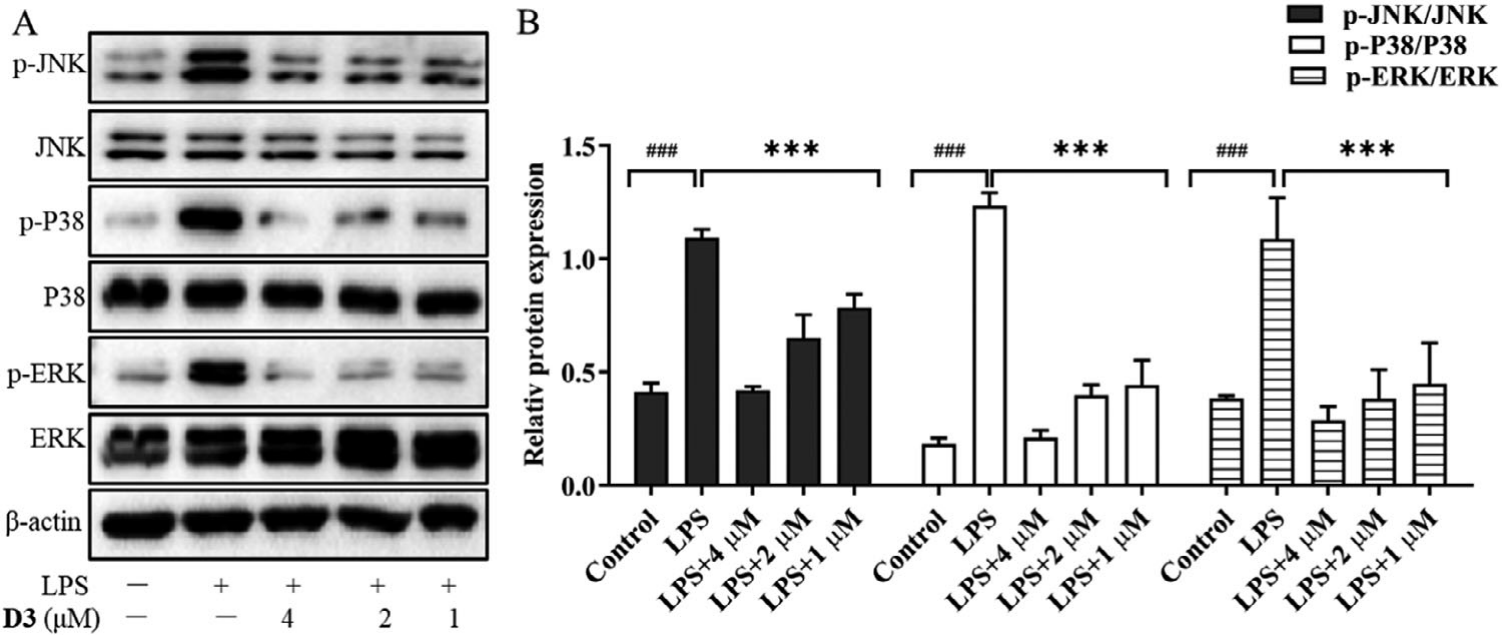
Compound **D3** inhibits LPS‐induced activation of MARK signaling pathway. All data are presented as mean ± standard deviation (SD), *n* = 3 independent biological replicates. ^###^ *p* < 0.001 vs Control; ***p < 0.001 versus compound **D3** treatment group.

The mitogen‐activated protein kinases (MAPK) are a family of signal transduction proteins that include extracellular signal‐regulated kinase (ERK), c‐Jun N‐terminal kinases (JNK), and the p38 isoform (p38), which are classical pathways that modulate immune‐mediated inflammatory responses and cellular activities involved in cell survival and apoptosis through the corresponding signaling cascades. Activated MAPKs modify the phosphorylation on the threonine/tyrosine motif, accelerating iNOS, COX‐2, and pro‐inflammatory cytokine expression in activated macrophages. To determine the effects of compound **D3** on the LPS‐induced phosphorylation of MAPKs, the expression of ERK, JNK, and p38 was examined. As expected, levels of phosphorylation of p38, JNK, and ERK were increased after LPS‐stimulated for 30 min (Figure 6). Compound **D3** suppressed LPS‐induced phosphorylation of p38 and ERK in 2 µM, but had little effect on phosphorylation of JNK in RAW264.7 cells. These results suggest that the anti‐inflammatory activity of compound **D3** may be related to the inhibition of phosphorylation of ERK and p38 proteins.

### 2.7 In Vivo Efficacy of Compound 3

**FIGURE 7:**
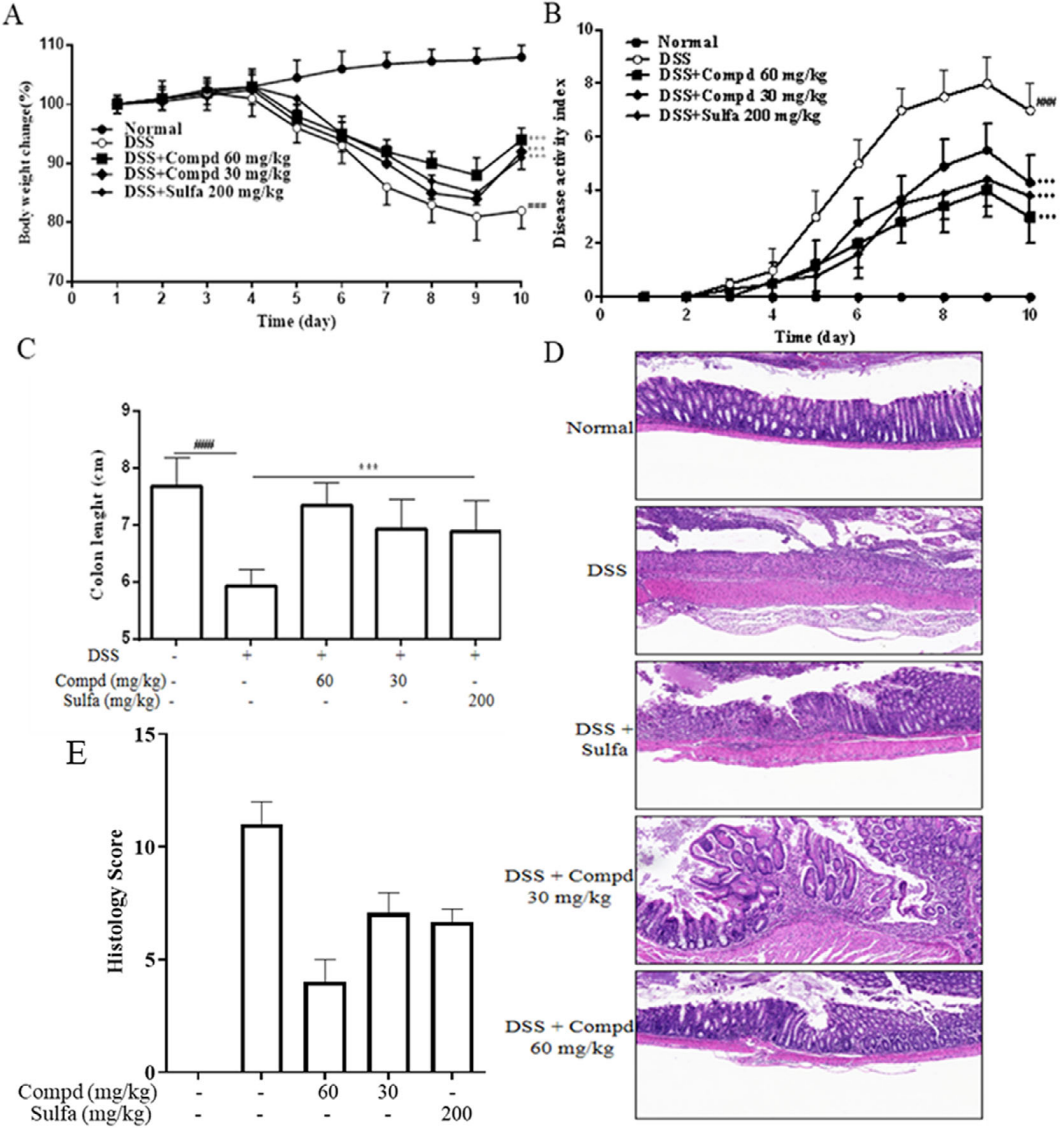
Therapeutic effect of Compound **D3** on DSS‐induced colitis in mice. (A) Record the daily weight of mice. (B) The daily DAI of the mice were calculated. (C) The colon length of mice was measured. (D) Hematoxylin and eosin (H & E) were used for pathological analysis of colon tissues. (E) Colonic histopathology score. ^###^ *p* < 0.001 compared with normal group; ***p < 0.001 compare with DSS group. Data are presented as mean ± SD (*n* = 5 mice per group).

Inflammatory bowel disease is a clinically complex syndrome with a multifaceted pathogenesis, necessitating further refinement and exploration of its mechanisms and treatment modalities. Among the widely used models for studying inflammatory bowel diseases, sodium dextran sulfate (DSS)‐induced colitis in mice stands out. DSS induces pathological manifestations such as diarrhea, rectal bleeding, and inflammatory cell infiltration by stimulating intestinal epithelial cells and compromising mucosal integrity. The present study utilized a 3% DSS aqueous solution to induce colitis in mice via daily oral gavage. Experimental findings (Figure 7) revealed that mice in the DSS‐induced model group exhibited a rapid decrease in body weight by the fourth day (Figure 7A), significantly increased rectal bleeding, reduced activity, and elevated Disease Activity Index (DAI) scores (Figure 7B). Upon completion of the experiment, mice were euthanized under anesthesia, and anatomical examination revealed that DSS‐induced mice had significantly shortened colons compared to the normal group. Furthermore, their colonic mucosal structure was damaged with crypt defects and extensive infiltration of inflammatory cells. During the colitis induction phase in this study, compound **D3** ameliorated clinical symptoms of DSS‐induced colitis including weight loss, rectal bleeding rate, colon length, and DAI scores (Figure 7C,D). Moreover, it partially restored the integrity of intestinal mucosal structure. In summary, these findings collectively demonstrate that compound **D3** can ameliorate DSS‐induced colitis. Based on inflammatory cell infiltration and crypt injury, tissue pathology scoring was performed using a blind method. DSS treatment significantly increased the histological score compared with the normal group, indicating severe mucosal injury and inflammatory infiltration. In contrast, administration of compound **D3** markedly reduced the histological score, demonstrating significant attenuation of tissue damage and preservation of colonic architecture (Figure 7E). These findings are consistent with the H&E staining observations and further confirm the therapeutic efficacy of compound **D3** in DSS‐induced colitis.

### 2.8 In Vivo Safety Evaluation of Compound **D3**

**FIGURE 8:**
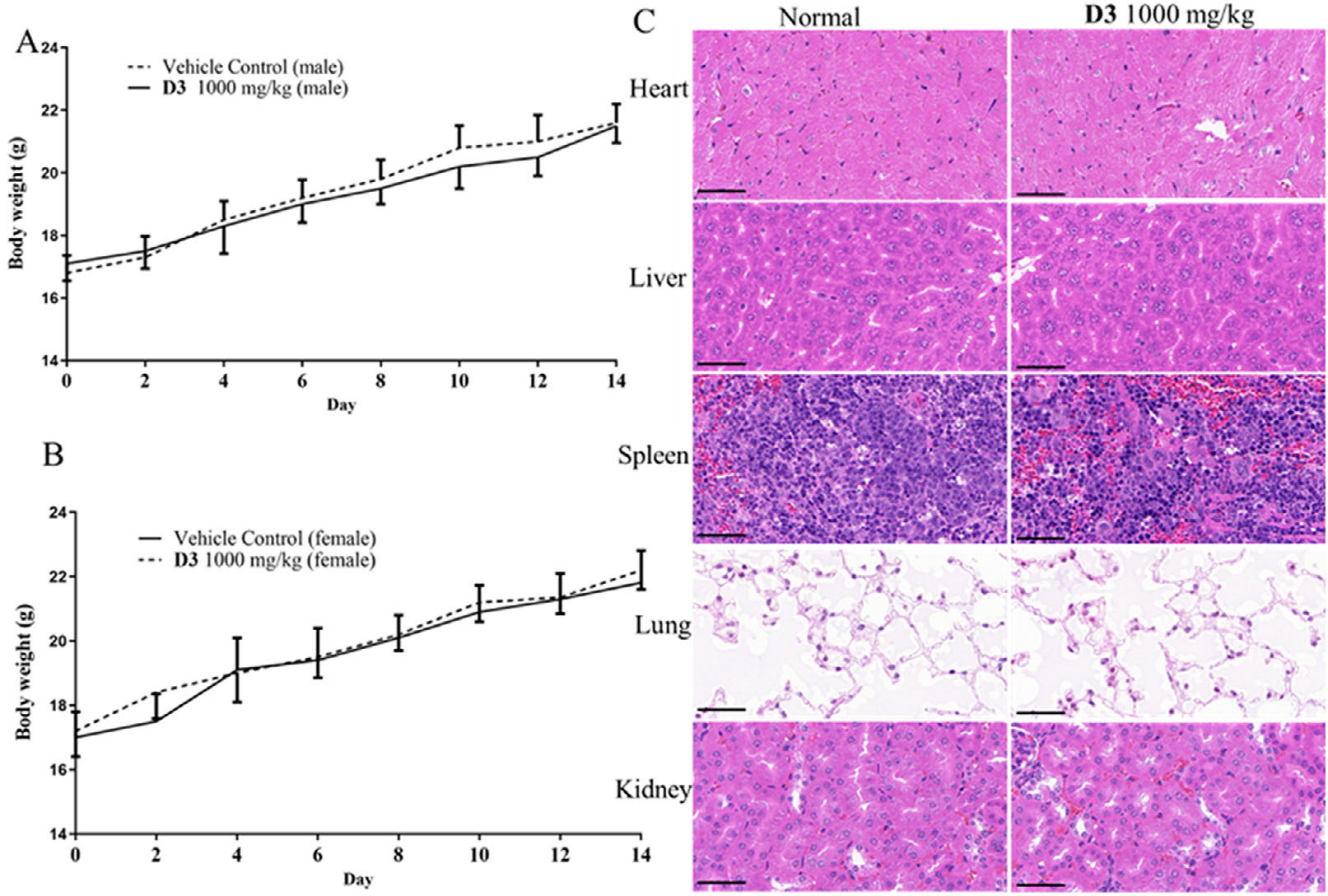
In vivo acute toxicity evaluation of compound **D3**. (A, B) Body weight changes in female/male mice following a single oral administration of D3 (1000 mg/kg) during the 14‐day observation period. (C) Pathological analysis (heart, liver, spleen, lungs, kidney). Data are presented as mean ± SD (*n* = 5 mice per group).

To further assess the preliminary in vivo safety profile of compound **D3**, an acute toxicity study was conducted in mice following a single oral administration of **D3** at a dose of 1000 mg/kg. Throughout the 14‐day observation period, no mortality, treatment‐related clinical signs, or abnormal behavioral responses were observed in either male or female mice. Throughout the entire experimental period, no significant difference in body weight was detected between the compound **D3** treatment group and the control group, indicating that **D3** did not have any adverse effects on normal growth (Figure 8A,B). At the end of the study, major organs were collected for histopathological examination. Histological analysis revealed normal tissue architecture in all examined organs from **D3**‐treated mice, no apparent pathological alterations were observed (Figure 8C). Collectively, these findings indicate that compound **D3** was well tolerated following acute administration at 1000 mg/kg and exhibited no detectable toxicological effects.

## 3 Discussion

Inflammatory bowel disease (IBD) is a refractory chronic inflammatory disorder with limited safe and effective clinical therapeutic options. Current first‐line clinical agents for IBD, including 5‐aminosalicylic acid derivatives, glucocorticoids, and immunosuppressants can only relieve partial inflammatory symptoms yet accompany prominent clinical drawbacks. Therefore, there is an urgent need to develop small molecule anti‐inflammatory lead compounds with low toxicity and oral administration for the discovery of IBD drugs.

Compared with currently available anti‐inflammatory therapies for IBD, compound **D3** demonstrated several noteworthy advantages. In the present study, compound **D3** exhibited potent inhibition of LPS‐induced NO production with an IC_50_ value of 1.2 µM and showed no apparent cytotoxicity at concentrations up to 50 µM, suggesting a favorable safety profile. Mechanistically, **D3** simultaneously inhibited NF‐κB and MAPK signaling pathways and suppressed the expression of iNOS and COX‐2. In vivo DSS‐induced acute colitis mouse model experiments further confirmed that oral **D3** significantly alleviated typical colitis manifestations including body weight loss, elevated DAI score, colon tissue shortening, and severe mucosal inflammatory infiltration with favorable therapeutic efficacy comparable to the positive control sulfasalazine. Preliminary acute toxicity test at a single oral dose of 1000 mg/kg demonstrated that **D3** possesses good preliminary in vivo safety, with no death, abnormal behavioral signs or pathological damage to major visceral organs within a 14‐day observation window.

Nevertheless, there are several inevitable limitations in the present study that need to be objectively addressed. First, although we confirmed that **D3** blocks LPS‐mediated TLR4 downstream inflammatory signaling, we have not yet confirmed the binding target of the compound. Second, our toxicological evaluation only covers a single‐dose acute toxicity experiment; systematic subchronic/chronic toxicity, reproductive toxicity, and organ‐specific long‐term toxic effects have not been characterized, and comprehensive ADME pharmacokinetic parameters of **D3** are still lacking, which hinders the further translational evaluation of this lead compound. Third, compound **D3** lacks comparison with various mainstream clinical IBD drugs (such as tofacitinib) in terms of in vivo efficacy and safety.

In response to the above limitations, clear directions have been developed for subsequent research. First, we use methods such as proteomics and genomics to determine the target of the compound. Second, a complete pharmacokinetic study of ADME and subchronic toxicity experiment in mice will be conducted to provide comprehensive preclinical toxicological and pharmacological data. Third, guided by the established SAR summarized in this manuscript, we will conduct further structural modification on the dithioacetal and aromatic linker substituents of the dihydropyrimidinone core to obtain derivatives with stronger in vivo colitis therapeutic activity and better pharmacokinetic properties, and perform parallel in vivo efficacy comparison with multiple commercial IBD clinical drugs to advance its translational research.

Collectively, this study provides a novel dual‐pathway anti‐inflammatory lead scaffold for the treatment of IBD, and systematically reveals the in vitro and in vivo anti‐inflammatory potential of dithioacetal‐functionalized dihydropyrimidinone derivatives. Although the current work has certain limitations in target confirmation and preclinical translational evaluation, it lays a solid foundation for the subsequent development of dihydropyrimidinone‐based oral small‐molecule IBD therapeutic agents.

## 4 Conclusion

In summary, based on active group splicing, a novel series of dihydropyridine derivatives as anti‐inflammatory agents were designed and synthesized. In vitro bioassays showed that title compounds could be alleviated the increase of LPS‐induced NO release in RAW264.7 cells. Compounds **D3**, **D19**, and **D30** exhibited excellently NO inhibitory activity (IC_50_ = 1.2 µM, 1.3 µM, and 1.21 µM, respectively). Western blot showed that compound **D3** decreased the expression of iNOS, COX‐2, and MAPKs, as well as activated NF‐кB in LPS‐stimulated RAW264.7 cells. Taken together, compound **D3** exerted its anti‐inflammatory activity through inhibition of NO generation as a result of inhibiting NF‐кB and MAPKs‐related inflammatory signaling pathways. These results could be very useful for the SAR study to develop new anti‐inflammatory drugs in the future.

### 4.1 Experimental Section

#### 4.1.1 General Methods for Chemistry

The ^1^H NMR and ^13^C NMR spectra were recorded on a VNMRS600 model spectrometer in DMSO solutions at room temperature with TMS as an internal standard. Chemical shifts (d) for H NMR and C NMR spectra were reported in parts per million to residual solvent protons. Melting points were measured on a Boetius micro melting point apparatus. EI‐MS were obtained on a Mariner System 5304 mass spectrometer.

#### 4.1.2 The Intermediate of Bromoethoxy‐Benzaldehyde (**A**)

This compound was prepared as previously reported [33]. A solution of hydroxybenzaldehyde (50 mM) and potassium carbonate (50 mM) in acetonitrile (30 mL) was stirred and heated to reflux for 30 min, then dibromoethane (200 mM) in acetonitrile (20 mL) was dropped to the solution over 1 h, and the mixture was stirred for 6 h. Then sodium hydroxide (30 mM) was added to the mixture and stirred for 30 min. The reaction solution was evaporated in vacuum and twice extracted with EtOAc. The organic phase was dried over anhydrous Na_2_SO_4_, filtered, and concentrated under the reduced pressure. The residue was purified by column chromatography on silica gel (PE/EtOAc = 5:1) to afford the intermediate A.

#### 4.1.3 4‐Bromoethoxy‐benzaldehyde (**A1**)

White powder, yield 23.2%, m.p. 55°C‐56°C; ^1^H NMR (600 MHz, CDCl_3_) *δ* 9.89 (s, 1H), 7.84 (d, *J* = 8.7 Hz, 2H), 7.01 (d, *J* = 8.6 Hz, 2H), 4.37 (t, *J* = 6.2 Hz, 2H), 3.66 (t, *J* = 6.2 Hz, 2H); ^13^C NMR (151 MHz, CDCl3) *δ* 190.92 (s), 163.19 (s), 132.22 (s), 130.66 (s), 115.06 (s), 68.13 (s), 28.67 (s).

#### 4.1.4 3‐Bromoethoxy‐benzaldehyde **(A2)**

Colorless viscous liquid, yield 27.2%; ^1^H NMR (600 MHz, CDCl_3_) *δ* 9.90 (s, 1H), 7.44 – 7.37 (m, 2H), 7.32 (s, 1H), 7.14 (dd, *J* = 7.9, 0.9 Hz, 1H), 4.29 (t, *J* = 6.1 Hz, 2H), 3.60 (t, *J* = 6.1 Hz, 2H); ^13^C NMR (151 MHz, CDCl_3_) *δ* 192.10 (s), 158.88 (s), 138.03 (s), 130.45 (s), 124.38 (s), 122.26 (s,), 113.07 (s), 68.21 (s), 29.01 (s).

#### 4.1.5 The Intermediate of Bromoethoxy‐dihydropyrimidinone (**B**)

A solution of the intermediate A (20 mM), urea or methylurea (22 mM), copper chloride dihydrate (0.5 mM), and acetylacetic ether (22 mmol) was heated to 90°C and stirred for 3 h, then dibromoethane (200 mM) in acetonitrile (20 mL) was dropped to the solution over 1 h, and the mixture was stirred for 6 h. The reaction mixture was washed three with water to obtain the residue. Then the residue was recrystallized from ethanol to obtain the intermediate B.

#### 4.1.6 5‐Ethoxycarbonyl‐6‐methyl‐4‐(4‐bromoethoxyphenyl)‐3,4‐dihydropyrimidin‐2(1H)‐one (**B1**)

White power, yield 79.0%, m.p. 160°C‐162°C; ^1^H NMR (600 MHz, DMSO) *δ* 9.16 (s, 1H), 7.68 (s, 1H), 7.16 (d, *J* = 8.6 Hz, 2H), 6.91 (d, *J* = 8.6 Hz, 2H), 5.10 (d, *J* = 3.1 Hz, 1H), 4.28 (t, *J* = 5.4 Hz, 2H), 3.98 (q, *J* = 7.1 Hz, 2H), 3.78 (t, *J* = 5.4 Hz, 2H), 2.24 (s, 3H), 1.10 (t, *J* = 7.1 Hz, 3H); ^13^C NMR (151 MHz, DMSO) δ 165.83 (s), 157.57 (s), 152.62 (s), 148.55 (s), 138.08 (s), 127.97 (s), 114.93 (s), 99.97 (s), 68.20 (s), 59.63 (s), 53.79 (s), 31.92 (s), 18.23 (s), 14.57 (s).

#### 4.1.7 5‐Ethoxycarbonyl‐6‐Methyl‐4‐(3‐Bromoethoxyphenyl)‐3,4‐Dihydropyrimidin‐2(1H)‐one (**B2**)

White power, yield 76.1%, m.p. 103°C–105°C; ^1^H NMR (600 MHz, DMSO) *δ* 7.90 (d, *J* = 3.8 Hz, 1H), 7.13 (d, *J* = 8.7 Hz, 2H), 6.90 (d, *J* = 8.7 Hz, 2H), 5.11 (d, *J* = 3.7 Hz, 1H), 4.30 – 4.27 (m, 2H), 4.05 – 4.00 (m, 2H), 3.79 – 3.76 (m, 2H), 3.09 (s, 3H), 2.48 (s, 3H), 1.12 (t, *J* = 7.1 Hz, 3H); ^13^C NMR (151 MHz, DMSO) *δ* 166.08 (s), 157.61 (s), 153.53 (s), 150.75 (s), 137.22 (s), 127.80 (s), 114.97 (s), 103.16 (s), 68.19 (s), 59.96 (s), 52.26 (s), 31.93 (s), 30.16 (s), 16.49 (s), 14.54 (s).

#### 4.1.8 5‐Ethoxycarbonyl‐6‐methyl‐4‐(4‐bromoethoxyphenyl)‐3‐methyl‐4‐hydropyrimidin‐2(1H)‐one (**B3**)

White power, yield 82.6%, m.p. 199‐201°C; ^1^H NMR (600 MHz, DMSO) *δ* 9.19 (s, 1H), 7.73 (s, 1H), 7.25 (t, *J* = 7.9 Hz, 1H), 6.85 (d, *J* = 8.0 Hz, 2H), 6.80 (s, 1H), 5.13 (d, *J* = 2.8 Hz, 1H), 4.27 (t, *J* = 6.7 Hz, 2H), 4.00 (q, *J* = 7.0 Hz, 2H), 3.80 (t, *J* = 5.3 Hz, 2H), 2.25 (s, 2H), 1.11 (t, *J* = 7.1 Hz, 3H); ^13^C NMR (151 MHz, DMSO) *δ* 165.81 (s), 158.37 (s), 152.65 (s), 148.97 (s), 146.93 (s), 130.12 (s), 119.28 (s), 113.60 (s), 113.22 (s), 99.54 (s), 68.14 (s), 59.70 (s), 54.18 (s), 31.91 (s), 18.26 (s), 14.58 (s).

#### 4.1.9 5‐Ethoxycarbonyl‐6‐methyl‐4‐(3‐bromoethoxyphenyl)‐3‐methyl‐4‐hydropyrimidin‐2(1H)‐one (**B4**)

White power, yield 70%, m.p. 170°C‐171°C; ^1^H NMR (600 MHz, DMSO) *δ* 7.97 (d, *J* = 3.6 Hz, 1H), 7.25 (t, *J* = 7.9 Hz, 1H), 6.87 – 6.82 (m, 2H), 6.78 (s, 1H), 5.15 (d, *J* = 3.6 Hz, 1H), 4.28 (dd, *J* = 5.7, 3.9 Hz, 2H), 4.05 (q, *J* = 7.0 Hz, 2H), 3.80 (t, *J* = 5.3 Hz, 2H), 3.10 (s, 3H), 2.49 (s, 3H), 1.14 (t, *J* = 7.1 Hz, 3H); ^13^C NMR (151 MHz, DMSO) δ 165.45 (s), 157.78 (s), 152.98 (s), 150.53 (s), 145.55 (s), 129.57 (s), 118.43 (s), 112.77 (s), 112.71 (s), 102.11 (s), 67.52 (s), 59.42 (s), 52.01 (s), 31.27 (s), 29.58 (s), 15.90 (s), 13.94 (s).

#### 4.1.10 The Intermediate of Dihydropyrimidinone with Aromatic Aldehyde (**C**)

A solution of the intermediate B (10 mM), K_2_CO_3_(10 mmol), and aromatic aldehyde containing hydroxyl group (10 mM) in acetonitrile (20 mL) was stirred and heated to reflux for 12 h. The reaction solution was evaporated in vacuum. The residue was recrystallized from 95% ethanol to obtain the intermediate C.

#### 4.1.11 5‐Ethoxycarbonyl‐6‐methyl‐4‐(4‐(2‐(4‐formylphenoxy)ethoxy)‐phenyl‐3,4‐dihydropyrimidin‐2(1H)‐one (**C1**)

White power, yield 54.3%, m.p. 150°C‐152°C; ^1^H NMR (600 MHz, DMSO) *δ* 9.88 (s, 1H), 9.16 (s, 1H), 7.88 (d, *J* = 8.7 Hz, 2H), 7.69 (s, 1H), 7.17 (dd, *J* = 8.6, 5.1 Hz, 4H), 6.94 (d, *J* = 8.7 Hz, 2H), 5.11 (d, *J* = 3.2 Hz, 1H), 4.46 – 4.40 (m, 2H), 4.35 – 4.31 (m, 2H), 3.98 (q, *J* = 7.1 Hz, 2H), 2.25 (s, 3H), 1.10 (t, *J* = 7.1 Hz, 3H); ^13^C NMR (151 MHz, DMSO) *δ* 191.32 (s), 165.36 (s), 163.29 (s), 157.44 (s), 152.15 (s), 148.06 (s), 137.43 (s), 131.82 (s), 129.80 (s), 127.48 (s), 115.00 (s), 114.35 (s), 99.52 (s), 66.88 (s), 66.12 (s), 59.15 (s), 53.33 (s), 17.76 (s), 14.10 (s).

#### 4.1.12 5‐Ethoxycarbonyl‐6‐methyl‐4‐(4‐(2‐(3‐formylphenoxy)ethoxy)‐phenyl)‐3,4‐dihydropyrimidin‐2(1H)‐one (**C2**)

White power, yield 55.7%, m.p. 134°C‐136°C; ^1^H NMR (600 MHz, DMSO) *δ* 9.98 (s, 1H), 9.16 (d, *J* = 1.2 Hz, 1H), 7.68 (d, *J* = 2.2 Hz, 1H), 7.53 (dd, *J* = 3.3, 2.5 Hz, 2H), 7.49 (d, *J* = 2.2 Hz, 1H), 7.35–7.31 (m, 1H), 7.17 (d, *J* = 8.6 Hz, 2H), 6.94 (d, *J* = 8.7 Hz, 2H), 5.11 (d, *J* = 3.2 Hz, 1H), 4.42–4.37 (m, 2H), 4.34–4.28 (m, 2H), 3.98 (q, *J* = 7.1 Hz, 2H), 2.25 (s, 3H), 1.10 (t, *J* = 7.1 Hz, 3H); ^13^C NMR (151 MHz, DMSO) δ 192.92 (s), 165.36 (s), 158.85 (s), 157.47 (s), 152.14 (s), 148.06 (s), 137.66 (s), 137.39 (s), 130.42 (s), 127.47 (s), 122.53 (s), 121.45 (s), 114.33 (s), 113.82 (s), 99.52 (s), 66.67 (s), 66.22 (s), 59.14 (s), 53.33 (s), 17.75 (s), 14.10 (s).

#### 4.1.13 5‐Ethoxycarbonyl‐6‐methyl‐4‐(3‐(2‐(4‐formylphenoxy)ethoxy)‐phenyl)‐3‐methyl‐4‐hydropyrimidin‐2(1H)‐one **(C3)**

White powder, yield 62.3%, m.p. 175°C‐177°C; ^1^H NMR (600 MHz, DMSO) *δ* 9.89 (s, 1H), 9.19 (s, 1H), 7.88 (d, *J* = 8.6 Hz, 2H), 7.73 (s, 1H), 7.26 (t, *J* = 7.9 Hz, 1H), 7.19 (d, *J* = 8.6 Hz, 2H), 6.89 (dd, *J* = 8.2, 1.9 Hz, 1H), 6.87 – 6.81 (m, 2H), 5.13 (d, *J* = 3.0 Hz, 1H), 4.45 (t, *J* = 4.2 Hz, 2H), 4.33 – 4.30 (m, 2H), 3.99 (q, *J* = 7.0 Hz, 2H), 2.25 (s, 3H), 1.10 (t, *J* = 7.0 Hz, 3H); ^13^C NMR (151 MHz, DMSO) *δ* 191.33 (s), 165.34 (s), 163.28 (s), 158.23 (s), 152.17 (s), 148.49 (s), 146.42 (s), 131.82 (s), 129.81 (s), 129.62 (s), 118.66 (s), 115.01 (s), 113.04 (s), 112.63 (s), 99.06 (s), 66.83 (s), 66.02 (s), 59.21 (s), 53.73 (s), 17.78 (s), 14.09 (s).

#### 4.1.14 5‐Ethoxycarbonyl‐6‐methyl‐4‐(3‐(2‐(3‐formylphenoxy)ethoxy)‐phenyl)‐3,4‐dihydropyrimidin‐2(1H)‐one (**C4**)

White powder, yield 66.0%, m.p. 158°C‐159°C; ^1^H NMR (600 MHz, DMSO) *δ* 9.99 (s, 1H), 9.19 (s, 1H), 7.74 (s, 1H), 7.57 – 7.52 (m, 2H), 7.50 (d, *J* = 2.2 Hz, 1H), 7.34 (dt, *J* = 6.3, 3.4 Hz, 1H), 7.26 (t, *J* = 7.9 Hz, 1H), 6.89 (dd, *J* = 8.1, 2.1 Hz, 1H), 6.86 – 6.81 (m, 2H), 5.14 (d, *J* = 3.2 Hz, 1H), 4.44 – 4.40 (m, 2H), 4.31 (dd, *J* = 7.5, 3.6 Hz, 2H), 4.00 (q, *J* = 7.1 Hz, 2H), 2.25 (s, 3H), 1.10 (t, *J* = 7.1 Hz, 3H); ^13^C NMR (151 MHz, DMSO) *δ* 192.92 (s), 165.35 (s), 158.85 (s), 158.27 (s), 152.18 (s), 148.49 (s), 146.42 (s), 137.67 (s), 130.42 (s), 129.62 (s), 122.56 (s), 121.48 (s), 118.64 (s), 113.80 (s), 113.03 (s), 112.64 (s), 99.07 (s), 66.63 (s), 66.13 (s), 59.22 (s), 53.75 (s), 17.79 (s), 14.09 (s).

#### 4.1.15 5‐Ethoxycarbonyl‐6‐methyl‐4‐(4‐(2‐(4‐formylphenoxy)ethoxy)‐phenyl)‐3‐methyl‐4‐hydropyrimidin‐2(1H)‐one (**C5**)

White powder, yield 54.8%, m.p. 135°C‐137°C; ^1^H NMR (600 MHz, DMSO) *δ* 9.88 (s, 1H), 7.91 (d, *J* = 3.7 Hz, 1H), 7.88 (d, *J* = 8.6 Hz, 2H), 7.16 (dd, *J* = 15.2, 8.5 Hz, 4H), 6.94 (d, *J* = 8.5 Hz, 2H), 5.12 (d, *J* = 3.5 Hz, 1H), 4.48 – 4.42 (m, 2H), 4.32 (d, *J* = 4.3 Hz, 2H), 4.03 (dd, *J* = 7.0, 2.3 Hz, 2H), 3.10 (s, 3H), 2.48 (s, 3H), 1.12 (t, *J* = 7.1 Hz, 3H); ^13^C NMR (151 MHz, DMSO) δ 191.31 (s), 165.60 (s), 163.28 (s), 157.47 (s), 153.06 (s), 150.25 (s), 136.57 (s), 131.82 (s), 129.80 (s), 127.31 (s), 114.99 (s), 114.38 (s), 102.71 (s), 66.86 (s), 66.10 (s), 59.47 (s), 51.81 (s), 29.68 (s), 16.01 (s), 14.06 (s).

#### 4.1.16 5‐Ethoxycarbonyl‐6‐methyl‐4‐(4‐(2‐(3‐formylphenoxy)ethoxy)‐phenyl)‐3‐methyl‐4‐hydropyrimidin‐2(1H)‐one (**C6**)

White powder, yield 47.7%, m.p. 75°C‐77°C; ^1^H NMR (600 MHz, DMSO) *δ* 9.98 (s, 1H), 7.91 (d, *J* = 3.5 Hz, 1H), 7.53 (d, *J* = 5.0 Hz, 2H), 7.48 (s, 1H), 7.32 (d, *J* = 5.2 Hz, 1H), 7.15 (d, *J* = 8.4 Hz, 2H), 6.93 (d, *J* = 8.4 Hz, 2H), 5.12 (d, *J* = 3.1 Hz, 1H), 4.39 (s, 2H), 4.32 (d, *J* = 3.8 Hz, 2H), 4.03 (dd, *J* = 12.8, 5.8 Hz, 2H), 3.10 (s, 3H), 2.48 (s, 3H), 1.12 (t, *J* = 7.1 Hz, 3H); ^13^C NMR (151 MHz, DMSO) *δ* 192.92 (s), 165.60 (s), 158.84 (s), 157.50 (s), 153.06 (s), 150.24 (s), 137.65 (s), 136.53 (s), 130.42 (s), 127.31 (s), 122.55 (s), 121.44 (s), 114.37 (s), 113.79 (s), 102.71 (s), 66.66 (s), 66.20 (s), 59.47 (s), 51.81 (s), 29.67 (s), 16.01 (s), 14.06 (s).

#### 4.1.17 5‐Ethoxycarbonyl‐6‐methyl‐4‐(3‐(2‐(4‐formylphenoxy)ethoxy)‐phenyl)‐3‐methyl‐4‐hydropyrimidin‐2(1H)‐one (**C7**)

White powder, yield 60.2%, m.p. 138°C‐140°C; ^1^H NMR (600 MHz, DMSO) *δ* 9.89 (s, 1H), 7.96 (d, *J* = 3.8 Hz, 1H), 7.88 (d, *J* = 8.7 Hz, 2H), 7.26 (t, *J* = 7.9 Hz, 1H), 7.19 (d, *J* = 8.7 Hz, 2H), 6.89 (dd, *J* = 8.1, 2.0 Hz, 1H), 6.83 (d, *J* = 7.7 Hz, 1H), 6.80 (s, 1H), 5.15 (d, *J* = 3.7 Hz, 1H), 4.48 – 4.42 (m, 2H), 4.34 – 4.29 (m, 2H), 4.08 – 4.01 (m, 2H), 3.09 (s, 3H), 2.48 (s, 3H), 1.12 (t, *J* = 7.1 Hz, 3H); ^13^C NMR (151 MHz, DMSO) *δ* 191.32 (s), 165.58 (s), 163.27 (s), 158.25 (s), 153.10 (s), 150.65 (s), 145.66 (s), 131.81 (s), 129.81 (s), 129.68 (s), 118.46 (s), 115.00 (s), 112.77 (s), 112.71 (s), 102.24 (s), 66.81 (s), 66.02 (s), 59.54 (s), 52.18 (s), 29.70 (s), 16.03 (s), 14.07 (s).

#### 4.1.18 5‐Ethoxycarbonyl‐6‐methyl‐4‐(3‐(2‐(3‐formylphenoxy)ethoxy)‐phenyl)‐3‐methyl‐4‐hydropyrimidin‐2(1H)‐one (**C8**)

White powder, yield 68.4%, m.p. 154‐156; ^1^H NMR (600 MHz, DMSO) *δ* 9.99 (s, 1H), 7.96 (d, *J* = 3.8 Hz, 1H), 7.54 (dd, *J* = 3.4, 2.2 Hz, 2H), 7.49 (d, *J* = 2.2 Hz, 1H), 7.34 (dt, *J* = 6.4, 3.4 Hz, 1H), 7.26 (t, *J* = 7.9 Hz, 1H), 6.89 (dd, *J* = 8.1, 2.1 Hz, 1H), 6.83 (d, *J* = 7.8 Hz, 1H), 6.80 (s, 1H), 5.15 (d, *J* = 3.7 Hz, 1H), 4.42 – 4.39 (m, 2H), 4.31 (dd, *J* = 7.2, 3.6 Hz, 2H), 4.07 – 4.01 (m, 2H), 3.09 (s, 3H), 2.49 (s, 3H), 1.12 (t, *J* = 7.1 Hz, 3H); ^13^C NMR (151 MHz, DMSO) *δ* 192.91 (s), 165.59 (s), 158.84 (s), 158.29 (s), 153.10 (s), 150.64 (s), 145.66 (s), 137.66 (s), 130.42 (s), 129.67 (s), 122.59 (s), 121.47 (s), 118.43 (s), 113.75 (s), 112.76 (s), 112.72 (s), 102.25 (s), 66.61 (s), 66.13 (s), 59.54 (s), 52.19 (s), 29.70 (s), 16.02 (s), 14.06 (s).

#### 4.1.19 5‐Ethoxycarbonyl‐6‐methyl‐4‐(4‐(2‐(3‐chloro‐4‐formylphenoxy)ethoxy)‐phenyl)‐3‐methyl‐4‐hydropyrimidin‐2(1H)‐one (**C9**)

White powder, yield 67.9%, m.p. 165°C‐167°C; ^1^H NMR (600 MHz, DMSO) *δ* 9.88 (s, 1H), 7.96 (s, 1H), 7.93–7.88 (m, 2H), 7.43 (d, *J* = 8.5 Hz, 1H), 7.15 (d, *J* = 8.3 Hz, 2H), 6.94 (d, *J* = 8.3 Hz, 2H), 5.12 (d, *J* = 3.5 Hz, 1H), 4.53 (d, *J* = 4.1 Hz, 2H), 4.36 (d, *J* = 4.0 Hz, 2H), 4.03 (qd, *J* = 7.0, 3.4 Hz, 2H), 3.10 (s, 3H), 2.48 (s, 3H), 1.13 (t, *J* = 7.0 Hz, 3H); ^13^C NMR (151 MHz, DMSO) *δ* 190.70 (s), 165.61 (s), 158.41 (s), 157.49 (s), 153.08 (s), 150.27 (s), 136.62 (s), 130.73 (s), 130.63 (s), 130.25 (s), 127.30 (s), 122.24 (s), 114.48 (s), 113.95 (s), 102.72 (s), 68.14 (s), 66.09 (s), 59.48 (s), 51.77 (s), 29.69 (s), 16.01 (s), 14.07 (s).

#### 4.1.20 5‐Ethoxycarbonyl‐6‐methyl‐4‐(4‐(2‐(2‐fluoro‐4‐formylphenoxy)ethoxy)‐phenyl)‐3,4‐dihydropyrimidin‐2(1H)‐one (**C10**)

White powder, yield 49.9%, m.p. 138°C‐140°C; ^1^H NMR (600 MHz, DMSO) *δ* 9.87 (s, 1H), 9.15 (s, 1H), 7.96 (s, 1H), 7.90 (d, *J* = 8.5 Hz, 1H), 7.67 (s, 1H), 7.44 (d, *J* = 8.5 Hz, 1H), 7.16 (d, *J* = 8.4 Hz, 2H), 6.95 (d, *J* = 8.4 Hz, 2H), 5.10 (s, 1H), 4.54 (s, 2H), 4.36 (s, 2H), 3.98 (q, *J* = 7.0 Hz, 2H), 2.24 (s, 3H), 1.10 (t, *J* = 7.1 Hz, 3H); ^13^C NMR (151 MHz, DMSO) *δ* 190.68 (s), 165.36 (s), 158.42 (s), 157.46 (s), 152.16 (s), 148.06 (s), 137.50 (s), 130.74 (s), 130.61 (s), 130.26 (s), 127.47 (s), 122.26 (s), 114.44 (s), 113.94 (s), 99.53 (s), 68.13 (s), 66.11 (s), 59.16 (s), 53.33 (s), 17.76 (s), 14.10 (s).

#### 4.1.21 5‐Ethoxycarbonyl‐6‐methyl‐4‐(4‐(2‐(2‐chloro‐4‐formyl‐phenoxy)ethoxy)‐phenyl)‐3,4‐dihydropyrimidin‐2(1H)‐one (**C11**)

White powder, yield 38.3%, m.p. 108°C‐110°C; ^1^H NMR (600 MHz, DMSO) *δ* 9.88 (s, 1H), 9.16 (s, 1H), 7.79 (d, *J* = 7.8 Hz, 1H), 7.75 – 7.63 (m, 2H), 7.46 (t, *J* = 7.6 Hz, 1H), 7.16 (d, *J* = 7.4 Hz, 2H), 6.94 (d, *J* = 7.4 Hz, 2H), 5.10 (s, 1H), 4.52 (s, 2H), 4.35 (s, 2H), 3.98 (d, *J* = 6.7 Hz, 2H), 2.25 (s, 3H), 1.10 (t, *J* = 6.4 Hz, 3H); ^13^C NMR (151 MHz, DMSO) *δ* 190.85 (s), 165.36 (s), 157.37 (s), 152.14 (s), 151.50 (s), 148.07 (s), 137.48 (s), 129.86 (s), 128.33 (s), 127.48 (s), 115.40 (s), 115.28 (s), 114.74 (s), 114.36 (s), 99.51 (s), 67.88 (s), 66.02 (s), 59.15 (s), 53.31 (s), 17.75 (s), 14.10 (s).

#### 4.1.22 The Title Compounds of Dihydropyrimidinones with Dithioacetal Group (**D**)

The synthesis of the compounds was referred to previous reports [34]. A solution of the intermediate C (2 mM), R^3^SH (4 mM), [(CH_2_)_3_SO_3_HMIM]HSO_4_ (0.2 mM) in CH_2_Cl_2_ (10 mL) was stirred and heated to 40°C for 6 h. The reaction solution was evaporated in vacuum, washed twice with water, filtered. The residue was purified by column chromatography on silica gel (PE:EtOAc = 1:1) to afford the title compound D.

#### 4.1.23 5‐Ethoxycarbonyl‐6‐methyl‐4‐(4‐(2‐(4‐bis((4‐fluorophenyl)thio)‐methyl)‐phenoxy)‐ethoxy)‐phenyl‐3,4‐dihydropyrimidin‐2(1H)‐one (**D1**)

White powder, yield 81.6%; m.p. 95°C‐97°C; ^1^H NMR (600 MHz, CDCl_3_): *δ* 8.30 (s, 1H), 7.33 – 7.27 (m, 4H), 7.24 (d, *J* = 8.2 Hz, 2H), 7.19 (d, *J* = 8.3 Hz, 2H), 6.93 (t, *J* = 8.5 Hz, 4H), 6.87 (d, *J* = 8.2 Hz, 2H), 6.81 (d, *J* = 8.3 Hz, 2H), 5.89 (s, 1H), 5.35 (s, 1H), 5.22(s, 1H), 4.27 (s, 4H), 4.07 (tq, *J* = 7.1, 3.2 Hz, 2H), 2.33 (s, 3H), 1.16 (t, *J* = 7.1 Hz, 3H); ^13^C NMR (151 MHz, CDCl3): *δ* 165.69 (s), 163.67 (s), 162.03 (s), 158.40 (d, J = 18 Hz), 153.50 (s), 146.05 (s), 136.71 (s), 135.72 (s), 135.67 (s), 131.81 (s), 129.28 (s), 129.11 (s), 127.90 (s), 116.02 (d, J = 21 Hz), 114.80 (s), 114.57 (s), 101.60 (s), 66.55 (s), 61.44 (s), 60.00 (s), 55.15 (s), 18.62 (s), 14.20 (s). HRMS (ESI) m/z for C_35_H_32_F_2_N_2_O_5_S_2_ [M+H]^+^ calcd 663.1793, found 663.1790.

#### 4.1.24 5‐Ethoxycarbonyl‐6‐methyl‐4‐(4‐(2‐(4‐bis((4‐chlorophenyl)thio)‐methyl)‐phenoxy)‐ethoxy)‐phenyl‐3,4‐dihydropyrimidin‐2(1H)‐one (**D2**)

White powder, yield 89.3%, m.p. 81°C‐82°C; ^1^H NMR (600 MHz, CDCl_3_): *δ* 8.38(s, 1H), 7.24 (dd, *J* = 8.5, 2.0 Hz, 8H), 7.20 (d, *J* = 8.6 Hz, 4H), 6.89 – 6.85 (m, 2H), 6.85 – 6.81 (m, 2H), 5.90 (s, 1H), 5.35 (d, *J* = 2.8 Hz, 1H), 5.32 (s, 1H), 4.27 (s, 4H), 4.07 (qq, *J* = 6.6, 3.7 Hz, 2H), 2.33 (s, 3H), 1.17 (t, *J* = 7.1 Hz, 3H); ^13^C NMR (151 MHz, CDCl_3_) *δ* 165.73 (s), 158.50 (s), 158.24 (s), 153.66 (s), 146.17 (s), 136.71 (s), 134.22 (s), 134.08 (s), 132.65 (s), 131.31 (s), 129.15 (s), 129.06 (s), 127.92 (s), 114.74 (s), 114.65 (s), 101.52 (s), 66.51 (s), 66.48 (s), 60.08 (s), 60.03 (s), 55.10 (s), 18.63 (s), 14.23 (s). HRMS (ESI) m/z for C_35_H_32_Cl_2_N_2_O_5_S_2_ [M+Na]^+^ calcd 717.1022, found 717.1023.

#### 4.1.25 5‐Ethoxycarbonyl‐6‐methyl‐4‐(4‐(2‐(4‐bis((2‐allyl)thio)‐methyl)‐phenoxy)‐ethoxy)‐phenyl‐3,4‐dihydropyrimidin‐2(1H)‐one (**D3**)

White powder, yield 92.6%, m.p. 78°C‐80°C; ^1^H NMR (600 MHz, CDCl_3_): *δ* 8.57 (s, 1H), 7.40 – 7.31 (m, 2H),7.24 (d, *J* = 8.1 Hz, 2H), 6.94 – 6.82 (m, 4H), 6.04 (d, *J* = 3.0 Hz, 1H), 5.86 – 5.70 (m, 2H), 5.34 (d, *J* = 2.7 Hz, 1H), 5.09 (q, *J* = 10.6, 9.6 Hz, 4H), 4.75 (s, 1H), 4.29 (s, 4H), 4.07 (ddt, *J* = 11.4, 7.6, 4.0 Hz, 2H), 3.26 (dd, J = 13.7, 7.1 Hz, 2H), 3.03 (dd, *J* = 13.7, 7.1 Hz, 2H), 2.32 (s, 3H), 1.17 (t, *J* = 7.0 Hz, 3H); ^13^C NMR (151 MHz, CDCl_3_): *δ* 165.73 (s), 158.26 (s), 158.21 (s), 153.71 (s), 146.20 (s), 136.66 (s), 133.85 (s), 132.31 (s), 129.32 (s), 127.90 (s), 117.57 (s), 114.73 (s), 114.66 (s), 101.50 (s), 66.51 (s), 66.49 (s), 60.01 (s), 55.07 (s), 49.78 (s), 35.28 (s), 18.59 (s), 14.22 (s). HRMS (ESI) m/z for C_29_H_34_N_2_O_5_S_2_ [M+H]^+^ calcd 555.1982, found 555.1982.

#### 4.1.26 5‐Ethoxycarbonyl‐6‐methyl‐4‐(4‐(2‐(4‐bis((2‐hydroxyethyl)thio‐methyl)‐phenoxy)‐ethoxy)‐phenyl‐3,4‐dihydropyrimidin‐2(1H)‐one (**D4**)

White powder, yield 78.2%, m.p. 115°C‐117°C; ^1^H NMR (600 MHz, DMSO‐*d_6_*): δ 9.14 (d, *J* = 2.1 Hz, 1H), 7.69 (dd, *J* = 3.5, 2.0 Hz, 1H), 7.35 – 7.29 (m, 2H), 7.22 (t, *J* = 7.9 Hz, 1H), 6.94 – 6.90 (m, 2H), 6.84 (ddd, *J* = 8.2, 2.6, 0.9 Hz, 1H), 6.82 – 6.78 (m, 2H), 5.17 (s, 1H), 5.10 (d, *J* = 3.4 Hz, 1H), 4.77 (t, *J* = 5.5 Hz, 2H), 4.31 – 4.20 (m, 4H), 3.96 (q, *J* = 7.1 Hz, 2H), 3.46 (tdd, *J* = 6.7, 5.4, 1.3 Hz, 4H), 2.60 (dt, J = 13.6, 6.7 Hz, 2H), 2.52 – 2.47 (m, 2H), 2.21 (s, 3H), 1.06 (t, *J* = 7.1 Hz, 3H);^13^C NMR (151 MHz, DMSO‐d_6_): δ 165.84 (s), 158.76 (s), 158.18 (s), 152.68 (s), 148.96 (s), 146.87 (s), 133.56 (s), 130.11 (s), 129.32 (s), 119.07 (s), 114.76 (s), 113.47 (s), 113.12 (s), 99.57 (s), 66.76 (s), 66.65 (s), 61.08 (s), 59.73 (s), 54.23 (s), 52.21 (s), 34.88 (s), 18.28 (s), 14.59 (s). HRMS (ESI) m/z for C_27_H_34_N_2_O_7_S_2_ [M+Na]^+^ calcd 585.1700, found 585.1703.

#### 4.1.27 5‐Ethoxycarbonyl‐6‐methyl‐4‐(4‐(2‐(4‐bis((2‐hydroxerthyl)thio‐methyl)‐phenoxy)‐ethoxy)‐phenyl‐3,4‐dihydropyrimidin‐2(1H)‐one (**D5**)

White powder, yield 93.8%, m.p. 148°C‐150°C; ^1^H NMR (600 MHz, DMSO‐*d_6_*): δ 9.14 (s, 1H), 7.67 (s, 1H), 7.35(d, *J* = 8.3 Hz, 2H), 7.15 (d, *J* = 8.3 Hz, 2H), 6.93 (t, *J* = 9.1 Hz, 4H), 5.20 (s, 1H), 5.10 (s, 1H), 4.80 (t, *J* = 5.5 Hz, 2H), 4.28 (s, 4H), 3.98 (q, *J* = 7.1 Hz, 2H), 3.52 – 3.46 (m, 4H), 2.63 (dt, *J* = 13.5, 6.9 Hz, 2H), 2.52 (d, *J* = 6.7 Hz, 2H), 2.24 (s, 3H), 1.10 (t, *J* = 7.1 Hz, 3H); ^13^C NMR (151 MHz, DMSO‐d_6_): *δ* 165.85 (s), 158.19 (s), 157.98 (s), 152.62 (s), 148.51 (s), 137.80 (s), 133.55 (s), 129.30 (s), 127.95 (s), 114.79 (s), 114.76 (s), 100.02 (s), 66.82 (s), 66.77 (s), 61.07 (s), 59.65 (s), 53.81 (s), 52.22 (s), 34.87 (s), 18.23 (s), 14.58 (s). HRMS (ESI) m/z for C_27_H_34_N_2_O_7_S_2_ [M+Na]^+^ calcd 585.1700, found 585.1703.

#### 4.1.28 5‐Ethoxycarbonyl‐6‐methyl‐4‐(4‐(2‐(3‐bis((2‐hydroxyethyl)thio‐methyl)‐phenoxy)‐ethoxy)‐phenyl‐3,4‐dihydropyrimidin‐2(1H)‐one (**D6**)

White powder, yield 82.7%, m.p. 133°C‐135°C; ^1^H NMR (600 MHz, DMSO‐*d_6_*): *δ* 9.15 (s, 1H), 7.71 – 7.65 (m, 1H), 7.27 (t, *J* = 8.1 Hz, 1H), 7.17 (d, *J* = 8.6 Hz, 2H), 7.06 – 7.00 (m, 2H), 6.94 (d, *J* = 8.7 Hz, 2H), 6.90 (dd, *J* = 8.2, 2.4 Hz, 1H), 5.20 (s, 1H), 5.11 (d, *J* = 3.3 Hz, 1H), 4.81 (t, *J* = 5.5 Hz, 2H), 4.29 (s, 4H), 3.98 (q, *J* = 7.0 Hz, 2H), 3.51 (q, *J* = 5.7 Hz, 4H), 2.66 (dt, *J* = 13.4, 6.8 Hz, 2H), 2.55 (dt, *J* = 13.5, 6.9 Hz, 2H), 2.25 (s, 3H), 1.10 (t, *J* = 7.0 Hz, 3H); ^13^C NMR (151 MHz, DMSO‐d_6_): *δ* 165.85 (s), 158.69 (s), 157.99 (s), 152.64 (s), 148.51 (s), 143.16 (s), 137.83 (s), 130.06 (s), 127.96 (s), 120.52 (s), 114.79 (s), 114.37 (s), 113.96 (s), 100.03 (s), 66.79 (s), 61.06 (s), 59.65 (s), 53.83 (s), 52.68 (s), 34.98 (s), 18.24 (s), 14.58 (s). HRMS (ESI) m/z for C_27_H_34_N_2_O_7_S_2_ [M+Na]^+^ calcd 585.1700, found 585.1699.

#### 4.1.29 5‐Ethoxycarbonyl‐6‐methyl‐4‐(4‐(2‐(3‐bis((2‐hydroxerthyl)thio‐methyl)‐phenoxy)‐ethoxy)‐phenyl‐3‐methyl‐4‐hydropyrimidin‐2(1H)‐one (**D7**)

White powder, yield 89.3%, m.p. 98°C‐100°C; ^1^H NMR (600 MHz, DMSO‐*d_6_*): *δ* 7.91 (d, *J* = 3.9 Hz, 1H), 7.36 (d, *J* = 8.6 Hz, 2H), 7.15 (d, *J* = 8.7 Hz, 2H), 6.94 (dd, *J* = 12.5, 8.7 Hz, 4H), 5.21 (s, 1H), 5.13 (d, *J* = 3.8 Hz, 1H), 4.82 (t, *J* = 5.4 Hz, 2H), 4.29 (s, 4H), 4.03 (qd, *J* = 7.0, 1.9 Hz, 2H), 3.50 (q, *J* = 6.0 Hz, 4H), 3.10 (s, 3H), 2.64 (dt, *J* = 13.6, 6.9 Hz, 2H), 2.56 – 2.51 (m, 2H), 2.49 (s, 3H), 1.13 (t, *J* = 7.1 Hz, 3H); ^13^C NMR (151 MHz, DMSO‐d_6_): *δ* 166.09 (s), 158.18 (s), 158.02 (s), 153.56 (s), 150.71 (s), 136.96 (s), 133.56 (s), 129.31 (s), 127.79 (s), 114.84 (s), 114.76 (s), 103.22 (s), 66.81 (s), 66.76 (s), 61.08 (s), 59.97 (s), 52.30 (s), 52.23 (s), 34.88 (s), 30.17 (s), 16.50 (s), 14.55 (s). HRMS (ESI) m/z for C_28_H_36_N_2_O_7_S_2_ [M+Na]^+^ calcd 599.1856, found 599.1856.

#### 4.1.30 5‐Ethoxycarbonyl‐6‐methyl‐4‐(3‐(2‐(4‐bis((2‐hydroxyethyl)thio)‐methyl)‐phenoxy)‐ethoxy)‐phenyl‐3‐methyl‐4‐hydropyrimidin‐2(1H)‐one (**D8**)

White powder, yield 87.6%, m.p. 102°C‐104°C; ^1^H NMR (600 MHz, CDCl_3_): *δ* 7.39 – 7.34 (m, 2H), 7.20 (t, *J* = 7.9 Hz, 1H), 6.91 – 6.87 (m, 2H), 6.86 – 6.79 (m, 3H), 5.98 (d, *J* = 3.4 Hz, 1H), 5.34 (d, *J* = 3.4 Hz, 1H), 5.06 (s, 1H), 4.27 (dq, *J* = 4.9, 2.1 Hz, 4H), 4.10 (q, *J* = 7.1 Hz, 2H), 3.70 (dt, *J* = 7.6, 4.5 Hz, 4H), 3.19 (s, 3H), 2.80 (dtd, *J* = 14.1, 5.9, 2.4 Hz, 2H), 2.73 – 2.63 (m, 4H), 2.49 (s, 3H), 1.18 (t, *J* = 7.1 Hz, 3H); ^13^C NMR (151 MHz, CDCl_3_): *δ* 166.09 (s), 158.84 (s), 158.40 (s), 154.06 (s), 149.43 (s), 145.03 (s), 132.53 (s), 129.79 (s), 128.97 (s), 118.98 (s), 114.84 (s), 113.44 (s), 113.17 (s), 104.04 (s), 66.56 (s), 66.45 (s), 61.36 (s), 60.27 (s), 53.72 (s), 52.53 (s), 35.46 (s), 30.37 (s), 16.59 (s), 14.22 (s). HRMS (ESI) m/z for C_28_H_36_N_2_O_7_S_2_ [M+H]^+^ calcd 577.2037, found 577.2039.

#### 4.1.31 5‐Ethoxycarbonyl‐6‐methyl‐4‐(4‐(2‐(4‐bis((4‐fluorophenyl)thio)‐methyl)‐phenoxy)‐ethoxy)‐phenyl‐3‐methyl‐4‐hydropyrimidin‐2(1H)‐one (**D9**)

White powder, yield 72.7%, m.p. 111°C‐113°C; ^1^H NMR (600 MHz, CDCl_3_): *δ* 7.34 – 7.28 (m, 4H), 7.18 (ddd, *J* = 9.0, 5.6, 2.5 Hz, 4H), 6.96 – 6.90 (m, 4H), 6.88 – 6.84 (m, 2H), 6.83 – 6.80 (m, 2H), 5.79 (d, *J* = 3.3 Hz, 1H), 5.34 (d, *J* = 3.3 Hz, 1H), 5.22 (s, 1H), 4.27 (s, 4H), 4.10 (q, *J* = 7.2 Hz, 2H), 3.22 (s, 3H), 2.50 (s, 3H), 1.19 (t, *J* = 7.1 Hz, 3H); ^13^C NMR (151 MHz, CDCl_3_): *δ* 166.14 (s), 163.66 (s), 162.01 (s), 158.37 (d, *J* = 33 Hz), 153.95 (s), 149.02 (s), 136.32 (s), 135.74 (s), 135.69 (s), 131.76 (s), 129.21 (s), 129.11 (s), 127.55 (s), 116.04 (d, *J* = 21 Hz), 114.76 (s), 114.55 (s), 104.46 (s), 66.51 (s), 61.40 (s), 60.19 (s), 53.39 (s), 30.31 (s), 16.57 (s), 14.22 (s). HRMS (ESI) m/z for C_36_H_34_F_2_N_2_O_5_S_2_ [M+H]^+^ calcd 677.1950, found 677.1952.

#### 4.1.32 5‐Ethoxycarbonyl‐6‐methyl‐4‐(4‐(2‐(4‐bis((4‐chlorophenyl)thio)‐methyl)‐phenoxy)‐ethoxy)‐phenyl‐3‐methyl‐4‐hydropyrimidin‐2(1H)‐one (**D10**)

White powder, yield 71.3%, m.p. 140°C‐142°C; ^1^H NMR (600 MHz, CDCl_3_): *δ* 7.26 – 7.23 (m, 6H), 7.22 – 7.16 (m, 6H), 6.88 – 6.82 (m, 4H), 5.49 (d, *J* = 3.2 Hz, 1H), 5.34 (d, *J* = 3.2 Hz, 1H), 5.32 (s, 1H), 4.28 (s, 4H), 4.10 (q, *J* = 7.1 Hz, 2H), 3.24 (s, 3H), 2.51 (s, 3H), 1.19 (t, *J* = 7.1 Hz, 3H); ^13^C NMR (151 MHz, CDCl_3_): *δ* 166.13 (s), 158.50 (s), 158.15 (s), 153.93 (s), 149.01 (s), 136.31 (s), 134.21 (s), 134.08 (s), 132.65 (s), 131.33 (s), 129.15 (s), 129.05 (s), 127.55 (s), 114.76 (s), 114.66 (s), 104.46 (s), 66.51 (s), 66.50 (s), 60.20 (s), 60.09 (s), 53.41 (s), 30.32 (s), 16.58 (s), 14.22 (s). HRMS (ESI) m/z for C_36_H_34_Cl_2_N_2_O_5_S_2_ [M+Na]^+^ calcd 731.1178, found 731.1177.

#### 4.1.33 5‐Ethoxycarbonyl‐6‐methyl‐4‐(3‐(2‐(4‐bis((4‐chlorophenyl)thio)‐methyl)‐phenoxy)‐ethoxy)‐phenyl‐3‐methyl‐4‐hydropyrimidin‐2(1H)‐one (**D11**)

White powder, yield 81.5%, m.p. 102°C‐104°C; ^1^H NMR (600 MHz, CDCl_3_): *δ* 7.26 – 7.23 (m, 6H), 7.23 – 7.18 (m, 5H), 6.86 (dd, *J* = 8.1, 6.6 Hz, 3H), 6.84 – 6.81 (m, 2H), 5.84 (d, *J* = 3.4 Hz, 1H), 5.36 (d, *J* = 3.4 Hz, 1H), 5.33 (s, 1H), 4.27 (s, 4H), 4.11 (q, *J* = 7.1 Hz, 2H), 3.21 (s, 3H), 2.50 (s, 3H), 1.19 (t, *J* = 7.1 Hz, 3H); ^13^C NMR (151 MHz, CDCl_3_): *δ* 166.07 (s), 158.87 (s), 158.50 (s), 153.95 (s), 149.47 (s), 145.09 (s), 134.22 (s), 134.08 (s), 132.67 (s), 131.35 (s), 129.81 (s), 129.16 (s), 129.06 (s), 119.02 (s), 114.66 (s), 113.43 (s), 113.12 (s), 104.00 (s), 66.52 (s), 66.41 (s), 60.24 (s), 60.09 (s), 53.79 (s), 30.35 (s), 16.61 (s), 14.24 (s). HRMS (ESI) m/z for C_36_H_34_Cl_2_N_2_O_5_S_2_ [M+Na]^+^ calcd 731.1178, found 731.1175.

#### 4.1.34 5‐Ethoxycarbonyl‐6‐methyl‐4‐(3‐(2‐(3‐bis((2‐hydroxyethyl)thio)‐methyl)‐phenoxy)‐ethoxy)‐phenyl‐3,4‐dihydropyrimidin‐2(1H)‐one (**D12**)

White powder, yield 88.6%, m.p. 143°C‐145°C; ^1^H NMR (600 MHz, DMSO‐*d_6_*): *δ* 9.17 (s, 1H), 7.72 (t, *J* = 2.8 Hz, 1H), 7.27 (dt, *J* = 12.1, 7.9 Hz, 2H), 7.06 – 7.01 (m, 2H), 6.91 – 6.88 (m, 2H), 6.85 – 6.83(m, 2H), 5.20 (s, 1H), 5.14 (d, *J* = 3.3 Hz, 1H), 4.82 (t, *J* = 5.5 Hz, 2H), 4.31 – 4.27(m, 4H), 4.00 (q, *J* = 7.1 Hz, 2H), 3.55 – 3.48 (m, 4H), 2.66 (dt, *J* = 13.5, 6.8 Hz, 2H), 2.55 (dt, *J* = 13.4, 6.9 Hz, 2H), 2.25 (s, 3H), 1.10 (t, *J* = 7.1 Hz, 3H); ^13^C NMR (151 MHz, DMSO‐d_6_): *δ* 165.84 (s), 158.77 (s), 158.68 (s), 152.67 (s), 148.95 (s), 146.89 (s), 143.18 (s), 130.10 (s), 130.06 (s), 120.53 (s), 119.08 (s), 114.37 (s), 113.95 (s), 113.49 (s), 113.12 (s), 99.57 (s), 66.74 (s), 66.67 (s), 61.07 (s), 59.71 (s), 54.25 (s), 52.68 (s), 34.98 (s), 18.28 (s), 14.59 (s). HRMS (ESI) m/z for C_27_H_34_N_2_O_7_S_2_ [M+Na]^+^ calcd 585.1700, found 585.1700.

#### 4.1.35 5‐Ethoxycarbonyl‐6‐methyl‐4‐(4‐(2‐(3‐bis((2‐hydroxyethyl)thio)‐methyl)‐phenoxy)‐ethoxy)‐phenyl‐3‐methyl‐4‐hydropyrimidin‐2(1H)‐one (**D13**)

White powder, yield 94.7%, m.p. 67°C‐68°C; ^1^H NMR (600 MHz, CDCl_3_): *δ* 7.23 (t, *J* = 7.9 Hz, 1H), 7.19 – 7.14 (m, 2H), 7.05 – 7.00 (m, 2H), 6.89 – 6.82 (m, 3H), 6.10 (s, 1H), 5.32 (d, *J* = 3.3 Hz, 1H), 5.04 (s, 1H), 4.32 – 4.25 (m, 4H), 4.09 (q, *J* = 7.1 Hz, 2H), 3.70 (q, *J* = 5.7 Hz, 4H), 3.21 (s, 3H), 3.01 (s, 2H), 2.80 (dtd, *J* = 14.2, 5.9, 2.5 Hz, 2H), 2.66 (dt, *J* = 13.9, 5.9 Hz, 2H), 2.49 (s, 3H), 1.18 (t, *J* = 7.1 Hz, 3H); ^13^C NMR (151 MHz, CDCl_3_): *δ* 166.18 (s), 158.81 (s), 158.13 (s), 154.15 (s), 148.96 (s), 141.81 (s), 136.27 (s), 129.77 (s), 127.55 (s), 120.53 (s), 114.84 (s), 114.53 (s), 114.05 (s), 104.51 (s), 66.65 (s), 61.41 (s), 61.39 (s), 60.25 (s), 53.31 (s), 52.99 (s), 35.39 (s), 30.33 (s), 16.56 (s), 14.20 (s). HRMS (ESI) m/z for C_28_H_36_N_2_O_7_S_2_ [M+Na]^+^ calcd 599.1856, found 599.1859.

#### 4.1.36 5‐Ethoxycarbonyl‐6‐methyl‐4‐(3‐(2‐(3‐bis((4‐chlorophenyl)thio)‐methyl)‐phenoxy)‐ethoxy)‐phenyl‐3,4‐dihydropyrimidin‐2(1H)‐one (**D14**)

White powder, yield 85.8%, m.p. 65°C‐67°C; ^1^H NMR (600 MHz, DMSO‐*d_6_*): *δ* 9.18 (s, 1H), 7.72 (s, 1H), 7.41 (d, *J* = 8.5 Hz, 4H), 7.36 (d, *J* = 8.5 Hz, 4H), 7.25 (q, *J* = 8.0 Hz, 2H), 7.11 – 7.06 (m, 2H), 6.91 – 6.86 (m, 2H), 6.86 – 6.82 (m, 2H), 6.16 (s, 1H), 5.13 (d, *J* = 3.2 Hz, 1H), 4.25 (s, 4H), 3.99 (q, *J* = 7.1 Hz, 2H), 2.25 (s, 3H), 1.10 (t, *J* = 7.1 Hz, 3H); ^13^C NMR (151 MHz, DMSO‐*d_6_*): *δ* 165.84 (s), 158.76 (s), 158.72 (s), 152.70 (s), 148.96 (s), 146.91 (s), 140.50 (s), 133.38 (s), 133.20 (s), 132.87 (s), 130.23 (s), 130.09 (s), 129.40 (s), 120.76 (s), 119.12 (s), 114.89 (s), 114.54 (s), 113.53 (s), 113.11 (s), 99.60 (s), 66.79 (s), 66.60 (s), 59.71 (s), 56.79 (s), 54.29 (s), 18.30 (s), 14.58 (s). HRMS (ESI) m/z for C_35_H_32_Cl_2_N_2_O_5_S_2_ [M+Na]^+^ calcd 717.1022, found 717.1017.

#### 4.1.37 5‐Ethoxycarbonyl‐6‐methyl‐4‐(4‐(2‐(4‐bis((4‐methylphenyl)thio)‐methyl)‐phenoxy)‐ethoxy)‐phenyl‐3‐methyl‐4‐hydropyrimidin‐2(1H)‐one (**D15**)

White powder, yield 71.8%, m.p. 122°C‐123°C; ^1^H NMR (600 MHz, CDCl_3_): *δ* 7.25 (s, 2H), 7.22 (d, *J* = 7.7 Hz, 4H), 7.18 (d, *J* = 8.2 Hz, 2H), 7.04 (d, *J* = 7.7 Hz, 4H), 6.86 (d, *J* = 8.2 Hz, 2H), 6.82 (d, *J* = 8.3 Hz, 2H), 5.61 (d, *J* = 3.4 Hz, 1H), 5.34 (s, 1H), 5.30 (s, 1H), 4.27 (s, 4H), 4.10 (q, *J* = 7.1 Hz, 2H), 3.23 (s, 3H), 2.51 (s, 3H), 2.30 (s, 6H), 1.19 (t, *J* = 7.1 Hz, 3H); ^13^C NMR (151 MHz, CDCl_3_): *δ* 166.14 (s), 158.19 (s), 153.95 (s), 149.01 (s), 137.93 (s), 136.27 (s), 133.02 (s), 132.52 (s), 130.97 (s), 129.59 (s), 129.17 (s), 127.54 (s), 114.77 (s), 114.47 (s), 104.48 (s), 66.55 (s), 66.49 (s), 60.54 (s), 60.20 (s), 53.41 (s), 30.32 (s), 21.19 (s), 16.58 (s), 14.23 (s). HRMS (ESI) m/z for C_38_H_40_N_2_O_5_S_2_ [M+Na]^+^ calcd 691.2271, found 691.2269.

#### 4.1.38 5‐Ethoxycarbonyl‐6‐methyl‐4‐(3‐(2‐(4‐bis((4‐methylphenyl)thio)‐methyl)‐phenoxy)‐ethoxy)‐phenyl‐3‐methyl‐4‐hydropyrimidin‐2(1H)‐one (**D16**)

White powder, yield 77.2%, m.p. 101°C‐103°C; ^1^H NMR (600 MHz, CDCl_3_): *δ* 7.27 (d, *J* = 8.7 Hz, 2H), 7.23 – 7.19 (m, 5H), 7.04 (d, *J* = 7.9 Hz, 4H), 6.87 – 6.85 (m, 2H), 6.83 – 6.80 (m, 3H), 5.99 (d, *J* = 3.5 Hz, 1H), 5.36 (d, *J* = 3.3 Hz, 1H), 5.31 (s, 1H), 4.25 (s, 4H), 4.10 (q, *J* = 7.1 Hz, 2H), 3.20 (s, 3H), 2.50 (s, 3H), 2.29 (s, 6H), 1.18 (t, *J* = 7.1 Hz, 3H); ^13^C NMR (151 MHz, CDCl_3_): *δ* 166.08 (s), 158.89 (s), 158.19 (s), 154.02 (s), 149.52 (s), 145.10 (s), 137.93 (s), 133.01 (s), 132.54 (s), 130.99 (s), 129.78 (s), 129.60 (s), 129.18 (s), 118.97 (s), 114.47 (s), 113.44 (s), 113.13 (s), 103.99 (s), 66.49 (s), 66.46 (s), 60.54 (s), 60.23 (s), 53.74 (s), 30.35 (s), 21.20 (s), 16.60 (s), 14.25 (s). HRMS (ESI) m/z for C_38_H_40_N_2_O_5_S_2_ [M+Na]^+^ calcd 691.2271, found 691.2270.

#### 4.1.39 5‐Ethoxycarbonyl‐6‐methyl‐4‐(3‐(2‐(3‐bis((4‐methylphenyl)thio)‐methyl)‐phenoxy)‐ethoxy)‐phenyl‐3‐methyl‐4‐hydropyrimidin‐2(1H)‐one (**D17**)

White powder, yield 88.9%, m.p. 89°C‐91°C; ^1^H NMR (600 MHz, CDCl_3_): *δ* 7.25 – 7.20 (m, 5H), 7.16 (t, *J* = 7.9 Hz, 1H), 7.04 (d, *J* = 7.9 Hz, 4H), 6.96 – 6.90 (m, 2H), 6.87 (dd, *J* = 4.9, 2.8 Hz, 2H), 6.84 – 6.78 (m, 2H), 5.86 (d, *J* = 2.7 Hz, 1H), 5.36 (d, *J* = 3.3 Hz, 1H), 5.29 (s, 1H), 4.27 – 4.20 (m, 4H), 4.11 (q, *J* = 7.1 Hz, 2H), 3.21 (s, 3H), 2.50 (s, 3H), 2.29 (s, 6H), 1.18 (t, *J* = 7.1 Hz, 3H); ^13^C NMR (151 MHz, CDCl_3_): *δ* 166.07 (s), 158.93 (s), 158.61 (s), 153.97 (s), 149.50 (s), 145.07 (s), 141.58 (s), 138.07 (s), 133.11 (s), 130.83 (s), 129.80 (s), 129.63 (s), 129.42 (s), 120.78 (s), 118.96 (s), 114.78 (s), 113.67 (s), 113.46 (s), 113.15 (s), 103.99 (s), 66.48 (s), 61.21 (s), 60.24 (s), 53.80 (s), 30.36 (s), 21.19 (s), 16.60 (s), 14.25 (s). HRMS (ESI) m/z for C_38_H_40_N_2_O_5_S_2_ [M+Na]^+^ calcd 691.2271, found 691.2271.

#### 4.1.40 5‐Ethoxycarbonyl‐6‐methyl‐4‐(4‐(2‐(4‐bis((4‐methylphenyl)thio)‐methyl)‐phenoxy)‐ethoxy)‐phenyl‐3,4‐dihydropyrimidin‐2(1H)‐one (**D18**)

White powder, yield 76.6%, m.p. 92°C‐93°C; ^1^H NMR (600 MHz, CDCl_3_): *δ* 8.60 (d, *J* = 2.1 Hz, 1H), 7.29 – 7.21 (m, 8H), 7.05 (d, *J* = 7.9 Hz, 4H), 6.88 (d, *J* = 8.7 Hz, 2H), 6.82 (d, *J* = 8.7 Hz, 2H), 6.09 (t, *J* = 2.5 Hz, 1H), 5.36 (d, *J* = 2.9 Hz, 1H), 5.32 (s, 1H), 4.30 – 4.23 (m, 4H), 4.08 (tq, *J* = 7.1, 3.3 Hz, 2H), 2.33 (s, 3H), 2.30 (s, 6H), 1.17 (t, *J* = 7.1 Hz, 3H); ^13^C NMR (151 MHz, CDCl_3_): *δ* 165.75 (s), 158.28 (s), 158.19 (s), 153.76 (s), 146.22 (s), 137.93 (s), 136.69 (s), 133.03 (s), 132.50 (s), 130.98 (s), 129.60 (s), 129.18 (s), 127.91 (s), 114.76 (s), 114.47 (s), 101.52 (s), 66.54 (s), 66.49 (s), 60.54 (s), 60.02 (s), 55.08 (s), 21.21 (s), 18.60 (s), 14.24 (s). HRMS (ESI) m/z for C_37_H_38_N_2_O_5_S_2_ [M+Na]^+^ calcd 677.2114, found 677.2117.

#### 4.1.41 5‐Ethoxycarbonyl‐6‐methyl‐4‐(4‐(2‐(3‐bis((4‐methylphenyl)thio)‐methyl)‐phenoxy)‐ethoxy)‐phenyl‐3,4‐dihydropyrimidin‐2(1H)‐one (**D19**)

White powder, yield 74.9%, m.p. 90°C‐92°C; ^1^H NMR (600 MHz, CDCl_3_): *δ* 8.68 (s, 1H), 7.24 – 7.22 (m, 6H), 7.14 (t, *J* = 7.9 Hz, 1H), 7.03 (d, *J* = 7.9 Hz, 4H), 6.94 – 6.90 (m, 2H), 6.89 – 6.85 (m, 2H), 6.80 – 6.77 (m, 1H), 6.15 – 6.10 (m, 1H), 5.34 (d, *J* = 2.8 Hz, 1H), 5.28 (s, 1H), 4.25 – 4.18 (m, 4H), 4.06 (tq, *J* = 7.1, 3.5 Hz, 2H), 2.32 (s, 3H), 2.28 (s, 6H), 1.16 (t, *J* = 7.1 Hz, 3H); ^13^C NMR (151 MHz, CDCl_3_): *δ* 164.69 (s), 157.55 (s), 157.25 (s), 152.77 (s), 145.20 (s), 140.49 (s), 137.01 (s), 135.63 (s), 132.07 (s), 129.77 (s), 128.57 (s), 128.36 (s), 126.85 (s), 119.70 (s), 113.73 (s), 113.70 (s), 112.65 (s), 100.45 (s), 65.49 (s), 65.43 (s), 60.14 (s), 58.95 (s), 54.01 (s), 20.14 (s), 17.52 (s), 13.19 (s). HRMS (ESI) m/z for C_37_H_38_N_2_O_5_S_2_ [M+Na]^+^ calcd 677.2114, found 677.2114.

#### 4.1.42 5‐Ethoxycarbonyl‐6‐methyl‐4‐(3‐(2‐(3‐bis((4‐methylphenyl)thio)‐methyl)‐phenoxy)‐ethoxy)‐phenyl‐3,4‐dihydropyrimidin‐2(1H)‐one (**D20**)

White powder, yield 94.9%, m.p. 61°C‐63°C; ^1^H NMR (600 MHz, CDCl_3_): *δ* 8.50 (s, 1H), 7.24 – 7.20 (m, 5H), 7.15 (t, *J* = 7.9 Hz, 1H), 7.04 (d, *J* = 8.0 Hz, 4H), 6.95 – 6.90 (m, 4H), 6.84 – 6.78 (m, 2H), 6.06 (s, 1H), 5.36 (d, *J* = 2.9 Hz, 1H), 5.29 (s, 1H), 4.25 – 4.18 (m, 4H), 4.07 (qt, *J* = 7.1, 3.4 Hz, 2H), 2.32 (s, 3H), 2.28 (s, 6H), 1.15 (t, *J* = 7.1 Hz, 3H); ^13^C NMR (151 MHz, CDCl_3_): *δ* 164.62 (s), 157.87 (s), 157.57 (s), 152.57 (s), 145.61 (s), 144.35 (s), 140.50 (s), 137.01 (s), 132.07 (s), 129.78 (s), 128.79 (s), 128.58 (s), 128.36 (s), 119.70 (s), 118.28 (s), 113.74 (s), 112.65 (s), 112.63 (s), 112.35 (s), 99.97 (s), 65.44 (s), 60.16 (s), 59.00 (s), 54.52 (s), 20.14 (s), 17.60 (s), 13.19 (s). HRMS (ESI) m/z for C_37_H_38_N_2_O_5_S_2_ [M+Na]^+^ calcd 677.2114, found 677.2110.

#### 4.1.43 5‐Ethoxycarbonyl‐6‐methyl‐4‐(3‐(2‐(4‐bis((4‐methylphenyl)thio)‐methyl)‐phenoxy)‐ethoxy)‐phenyl‐3,4‐dihydropyrimidin‐2(1H)‐one (**D21**)

White powder, yield 89.6%, m.p. 68°C‐70°C; ^1^H NMR (600 MHz, CDCl_3_): *δ* 8.61 (s, 1H), 7.22 (td, *J* = 12.9, 12.2, 7.8 Hz, 7H), 7.03 (d, *J* = 7.8 Hz, 4H), 6.91 (d, *J* = 7.7 Hz, 2H), 6.81 (dd, *J* = 9.4, 2.8 Hz, 3H), 6.19 (s, 1H), 5.35 (d, *J* = 2.8 Hz, 1H), 5.31 (s, 1H), 4.23 (s, 4H), 4.06 (dtt, *J* = 10.9, 7.1, 3.8 Hz, 2H), 2.31 (s, 3H), 2.28 (s, 6H), 1.14 (t, *J* = 7.1 Hz, 3H); ^13^C NMR (151 MHz, CDCl_3_): *δ* 164.62 (s), 157.84 (s), 157.15 (s), 152.71 (s), 145.64 (s), 144.35 (s), 136.87 (s), 131.96 (s), 131.43 (s), 129.94 (s), 128.77 (s), 128.55 (s), 128.12 (s), 118.28 (s), 113.41 (s), 112.61 (s), 112.30 (s), 99.95 (s), 65.42 (s), 59.49 (s), 58.99 (s), 54.45 (s), 20.15 (s), 17.57 (s), 13.19 (s). HRMS (ESI) m/z for C_37_H_38_N_2_O_5_S_2_ [M+Na]^+^ calcd 677.2114, found 677.2113.

#### 4.1.44 5‐Ethoxycarbonyl‐6‐methyl‐4‐(3‐(2‐(4‐bis((4‐chlorophenyl)thio)‐methyl)‐phenoxy)‐ethoxy)‐phenyl‐3,4‐dihydropyrimidin‐2(1H)‐one (**D22**)

White powder, yield 71.9%, m.p. 70°C‐72°C; ^1^H NMR (600 MHz, CDCl_3_): *δ* 8.77 (s, 1H), 7.25 – 7.22 (m, 7H), 7.20 – 7.18 (m, 4H), 6.93 – 6.89 (m, 2H), 6.84 – 6.79 (m, 3H), 6.40 (t, *J* = 2.5 Hz, 1H), 5.35 (d, *J* = 2.9 Hz, 1H), 5.33 (s, 1H), 4.24 (s, 4H), 4.06 (qt, *J* = 7.1, 3.8 Hz, 2H), 2.31 (s, 3H), 1.14 (t, *J* = 7.1 Hz, 3H); ^13^C NMR (151 MHz, CDCl_3_): *δ* 164.63 (s), 157.80 (s), 157.47 (s), 152.89 (s), 145.71 (s), 144.40 (s), 133.13 (s), 133.00 (s), 131.63 (s), 130.24 (s), 128.78 (s), 128.10 (s), 128.01 (s), 118.30 (s), 113.61 (s), 112.59 (s), 112.26 (s), 99.92 (s), 65.47 (s), 65.35 (s), 59.02 (s), 58.99 (s), 54.37 (s), 17.54 (s), 13.19 (s). HRMS (ESI) m/z for C_35_H_32_Cl_2_N_2_O_5_S_2_ [M+Na]^+^ calcd 717.1022, found 717.1027.

#### 4.1.45 5‐Ethoxycarbonyl‐6‐methyl‐4‐(3‐(2‐(3‐bis((4‐fluorophenyl)thio)‐methyl)‐phenoxy)‐ethoxy)‐phenyl‐3,4‐dihydropyrimidin‐2(1H)‐one (**D23**)

White powder, yield 88.7%, m.p. 57°C‐59°C; ^1^H NMR (600 MHz, CDCl_3_): *δ* 8.60 (s, 1H), 7.31 (dd, *J* = 8.4, 5.3 Hz, 4H), 7.22 (t, *J* = 8.0 Hz, 1H), 7.15 (t, *J* = 7.9 Hz, 1H), 6.93 (tt, *J* = 8.0, 3.2 Hz, 7H), 6.86 – 6.80 (m, 3H), 6.16 (t, *J* = 2.5 Hz, 1H), 5.37 (d, *J* = 2.8 Hz, 1H), 5.20 (s, 1H), 4.27 – 4.21 (m, 4H), 4.06 (qq, *J* = 7.0, 3.7 Hz, 2H), 2.32 (s, 3H), 1.15 (t, *J* = 7.1 Hz, 3H); ^13^C NMR (151 MHz, CDCl_3_): *δ* 164.63 (s), 162.67 (s), 161.02 (s), 157.76 (d, J = 25.1 Hz), 152.69 (s), 145.66 (s), 144.39 (s), 139.84 (s), 134.69 (d, J = 7.9 Hz), 128.79 (s), 128.49 (s), 128.08 (d, J = 3.3 Hz), 119.61 (s), 118.32 (s), 114.97 (d, J = 21.8 Hz), 113.71 (s), 112.83 (s), 112.60 (s), 112.32 (s), 99.95 (s), 65.49 (s), 65.39 (s), 60.97 (s), 58.99 (s), 54.47 (s), 17.57 (s), 13.18 (s). HRMS (ESI) m/z for C_35_H_32_F_2_N_2_O_5_S_2_ [M+Na]^+^ calcd 685.1613, found 685.1613.

#### 4.1.46 5‐Ethoxycarbonyl‐6‐methyl‐4‐(4‐(2‐(2‐chloro‐4‐bis((2‐hydroxyethyl)thio)‐methyl)‐phenoxy)‐ethoxy)‐phenyl‐3‐methyl‐4‐hydropyrimidin‐2(1H)‐one (**D24**)

White powder, yield 84.7%, m.p. 96°C‐98°C; ^1^H NMR (600 MHz, CDCl_3_): *δ* 7.47 (d, *J* = 2.3 Hz, 1H), 7.29 (dd, *J* = 8.5, 2.3 Hz, 1H), 7.16 (d, *J* = 8.5 Hz, 2H), 6.92 (d, *J* = 8.5 Hz, 1H), 6.87 – 6.83 (m, 2H), 6.20 (d, *J* = 3.4 Hz, 1H), 5.32 (d, *J* = 3.3 Hz, 1H), 5.07 (s, 1H), 4.36 – 4.30 (m, 4H), 4.09 (q, *J* = 7.1 Hz, 2H), 3.72 (q, *J* = 5.6 Hz, 4H), 3.20 (s, 3H), 3.05 – 3.04 (m, 2H), 2.83 – 2.78 (m, 2H), 2.64 (dt, *J* = 13.9, 5.9 Hz, 2H), 2.49 (s, 3H), 1.19 (t, *J* = 7.1 Hz, 3H); ^13^C NMR (151 MHz, CDCl_3_): *δ* 165.14 (s), 157.05 (s), 153.14 (s), 152.90 (s), 147.95 (s), 135.26 (s), 132.90 (s), 128.68 (s), 126.50 (s), 126.08 (s), 122.30 (s), 113.88 (s), 112.81 (s), 103.50 (s), 66.92 (s), 65.45 (s), 60.52 (s), 59.22 (s), 52.25 (s), 51.03 (s), 34.33 (s), 29.32 (s), 15.54 (s), 13.19 (s). HRMS (ESI) m/z for C_28_H_35_ClN_2_O_7_S_2_ [M+Na]^+^ calcd 633.1466, found 633.1469.

#### 4.1.47 5‐Ethoxycarbonyl‐6‐methyl‐4‐(4‐(2‐(2‐chloro‐4‐bis((4‐methylphenyl)thio)‐methyl)‐phenoxy)‐ethoxy)‐phenyl‐3‐methyl‐4‐hydropyrimidin‐2(1H)‐one (**D25**)

White powder, yield 76.3%, m.p. 125°C‐127°C; ^1^H NMR (600 MHz, CDCl_3_): *δ* 7.34 (d, *J* = 2.3 Hz, 1H), 7.22 (d, *J* = 7.9 Hz, 4H), 7.20 – 7.14 (m, 3H), 7.05 (d, *J* = 7.8 Hz, 4H), 6.90 – 6.86 (m, 2H), 6.84 (d, *J* = 8.4 Hz, 1H), 5.51 (d, *J* = 3.3 Hz, 1H), 5.34 (d, *J* = 3.2 Hz, 1H), 5.23 (s, 1H), 4.33 (s, 4H), 4.10 (q, *J* = 7.2 Hz, 2H), 3.24 (s, 3H), 2.51 (s, 3H), 2.30 (s, 6H), 1.19 (t, *J* = 7.1 Hz, 3H); ^13^C NMR (151 MHz, CDCl_3_): *δ* 166.13 (s), 158.16 (s), 153.96 (s), 153.71 (s), 149.03 (s), 138.27 (s), 136.34 (s), 133.74 (s), 133.26 (s), 130.44 (s), 129.92 (s), 129.68 (s), 127.53 (s), 127.22 (s), 123.00 (s), 114.89 (s), 113.46 (s), 104.48 (s), 67.86 (s), 66.47 (s), 60.20 (s), 60.10 (s), 53.39 (s), 30.32 (s), 21.20 (s), 16.58 (s), 14.22 (s). HRMS (ESI) m/z for C_38_H_39_ClN_2_O_5_S_2_ [M+Na]^+^ calcd 725.1881, found 725.1881.

#### 4.1.48 5‐Ethoxycarbonyl‐6‐methyl‐4‐(4‐(2‐(2‐chloro‐4‐bis((4‐chlorophenyl)thio)‐methyl)‐phenoxy)‐ethoxy)‐phenyl‐3‐methyl‐4‐hydropyrimidin‐2(1H)‐one (**D26**)

White powder, yield 71.5%, m.p. 175°C‐177°C; ^1^H NMR (600 MHz, CDCl_3_): *δ* 7.37 (d, *J* = 2.3 Hz, 1H), 7.27 – 7.20 (m, 8H), 7.18 (d, *J* = 8.6 Hz, 2H), 7.12 (dd, *J* = 8.5, 2.4 Hz, 1H), 6.91 – 6.85 (m, 2H), 6.85 (d, *J* = 8.5 Hz, 1H), 5.62 (d, *J* = 3.4 Hz, 1H), 5.34 (d, *J* = 3.2 Hz, 1H), 5.26 (s, 1H), 4.34 (s, 4H), 4.10 (q, *J* = 7.1 Hz, 2H), 3.23 (s, 3H), 2.51 (s, 3H), 1.19 (t, *J* = 7.1 Hz, 3H); ^13^C NMR (151 MHz, CDCl_3_): *δ* 165.07 (s), 157.10 (s), 153.05 (s), 152.83 (s), 147.97 (s), 135.34 (s), 133.51 (s), 133.25 (s), 131.52 (s), 131.13 (s), 128.82 (s), 128.13 (s), 126.51 (s), 126.12 (s), 122.31 (s), 113.87 (s), 112.42 (s), 103.43 (s), 66.83 (s), 65.41 (s), 59.17 (s), 58.63 (s), 52.42 (s), 29.28 (s), 15.55 (s), 13.19 (s). HRMS (ESI) m/z for C_36_H_33_Cl_3_N_2_O_5_S_2_ [M+Na]^+^ calcd 765.0789, found 765.0784.

#### 4.1.49 5‐Ethoxycarbonyl‐6‐methyl‐4‐(4‐(2‐(2‐chloro‐4‐bis((4‐fluorophenyl)thio)‐methyl)‐phenoxy)‐ethoxy)‐phenyl‐3‐methyl‐4‐hydropyrimidin‐2(1H)‐one (**D27**)

White powder, yield 70.8%, m.p. 136°C‐138°C; ^1^H NMR (600 MHz, CDCl_3_): *δ* 7.34 – 7.27 (m, 5H), 7.18 (d, *J* = 8.6 Hz, 2H), 7.07 (dd, *J* = 8.5, 2.4 Hz, 1H), 6.98 – 6.92 (m, 4H), 6.89 – 6.86 (m, 2H), 6.84 (d, *J* = 8.5 Hz, 1H), 5.71 (d, *J* = 3.3 Hz, 1H), 5.34 (d, *J* = 3.2 Hz, 1H), 5.16 (s, 1H), 4.33 (s, 4H), 4.10 (q, *J* = 7.1 Hz, 2H), 3.23 (s, 3H), 2.51 (s, 3H), 1.19 (t, *J* = 7.1 Hz, 3H); ^13^C NMR (151 MHz, CDCl_3_): *δ* 165.08 (s), 162.76 (s), 161.11 (s), 157.09 (s), 152.90 (s), 148.00 (s), 135.36 (s), 134.86 (d, J = 7.9 Hz), 131.94 (s), 128.81 (s), 127.72 (d, J = 3.3 Hz), 126.50 (s), 126.10 (s), 122.16 (s), 115.06 (d, J = 21.8 Hz), 113.85 (s), 112.37 (s), 103.43 (s), 66.83 (s), 65.43 (s), 59.85 (s), 59.16 (s), 52.36 (s), 29.27 (s), 15.54 (s), 13.18 (s). HRMS (ESI) m/z for C_36_H_33_ClF_2_N_2_O_5_S_2_ [M+Na]^+^ calcd 733.1380, found 733.1379.

#### 4.1.50 5‐Ethoxycarbonyl‐6‐methyl‐4‐(3‐(2‐(3‐bis((2‐hydroxyethyl)thio)‐methyl)‐phenoxy)‐ethoxy)‐phenyl‐3‐methyl‐4‐hydropyrimidin‐2(1H)‐one (**D28**)

White powder, yield 92.5%, m.p. 87°C‐88°C; ^1^H NMR (600 MHz, CDCl_3_): *δ* 7.22 (dt, *J* = 18.9, 7.9 Hz, 2H), 7.08 – 7.01 (m, 2H), 6.88 – 6.80 (m, 4H), 6.10 (d, *J* = 3.4 Hz, 1H), 5.34 (d, *J* = 3.3 Hz, 1H), 5.06 (s, 1H), 4.32 – 4.25 (m, 4H), 4.10 (q, *J* = 7.1 Hz, 2H), 3.70 (q, *J* = 5.6 Hz, 4H), 3.19 (s, 3H), 2.89 (q, *J* = 5.4 Hz, 2H), 2.80 (dt, *J* = 13.2, 6.8 Hz, 2H), 2.67 (dtd, *J* = 13.9, 5.9, 3.7 Hz, 2H), 2.49 (s, 3H), 1.18 (t, *J* = 7.1 Hz, 3H); ^13^C NMR (151 MHz, CDCl_3_): *δ* 165.09 (s), 157.84 (s), 157.79 (s), 153.11 (s), 148.41 (s), 144.00 (s), 140.81 (s), 128.76 (s), 119.51 (s), 117.97 (s), 113.61 (s), 113.04 (s), 112.54 (s), 112.14 (s), 103.03 (s), 65.64 (s), 65.52 (s), 60.43 (s), 59.26 (s), 52.65 (s), 51.98 (s), 34.43 (s), 29.35 (s), 15.56 (s), 13.20 (s). HRMS (ESI) m/z for C_28_H_36_N_2_O_7_S_2_ [M+Na]^+^ calcd 599.1856, found 599.1853.

#### 4.1.51 5‐Ethoxycarbonyl‐6‐methyl‐4‐(4‐(2‐(2‐chloro‐4‐bis((2‐allyl)thio)‐methyl)‐phenoxy)‐ethoxy)‐phenyl‐3,4‐dihydropyrimidin‐2(1H)‐one (**D29**)

White powder, yield 93.6%, m.p. 70°C‐72°C; ^1^H NMR (600 MHz, CDCl_3_): *δ* 8.67 (d, *J* = 2.1 Hz, 1H), 7.45 (d, *J* = 2.3 Hz, 1H), 7.29 (dd, *J* = 8.5, 2.4 Hz, 1H), 7.25 – 7.19 (m, 2H), 6.93 (d, *J* = 8.5 Hz, 1H), 6.90 – 6.85 (m, 2H), 6.18 (t, *J* = 2.6 Hz, 1H), 5.84 – 5.72 (m, 2H), 5.34 (d, *J* = 2.9 Hz, 1H), 5.14 – 5.06 (m, 4H), 4.70 (s, 1H), 4.34 (ddq, *J* = 8.0, 5.1, 3.3, 2.8 Hz, 4H), 4.10 – 4.03 (m, 2H), 3.26 (dd, *J* = 13.8, 7.2 Hz, 2H), 3.03 (dd, *J* = 13.8, 7.2 Hz, 2H), 2.31 (s, 3H), 1.17 (t, *J* = 7.1 Hz, 3H); ^13^C NMR (151 MHz, CDCl_3_): *δ* 165.74 (s), 158.21 (s), 153.84 (s), 153.81 (s), 146.26 (s), 136.76 (s), 133.67 (s), 130.04 (s), 127.88 (s), 127.41 (s), 123.24 (s), 117.79 (s), 114.86 (s), 113.81 (s), 101.48 (s), 67.93 (s), 66.44 (s), 60.00 (s), 55.00 (s), 49.29 (s), 35.34 (s), 18.57 (s), 14.23 (s). HRMS (ESI) m/z for C_29_H_33_ClN_2_O_5_S_2_ [M+Na]^+^ calcd 611.1412, found 611.1416.

#### 4.1.52 5‐Ethoxycarbonyl‐6‐methyl‐4‐(4‐(2‐(2‐fluoro‐4‐bis((2‐allyl)thio)‐methyl)‐phenoxy)‐ethoxy)‐phenyl‐3,4‐dihydropyrimidin‐2(1H)‐one (**D30**)

White powder, yield 94.3%, m.p. 78°C‐80°C; ^1^H NMR (600 MHz, CDCl_3_): *δ* 8.60 (d, *J* = 2.1 Hz, 1H), 7.24 – 7.20 (m, 3H), 7.12 (dd, *J* = 7.8, 2.0 Hz, 1H), 6.96 (t, *J* = 8.4 Hz, 1H), 6.89 – 6.83 (m, 2H), 6.08 (t, *J* = 2.6 Hz, 1H), 5.78 (ddt, J = 16.9, 9.6, 7.2 Hz, 2H), 5.34 (d, *J* = 2.8 Hz, 1H), 5.10 (dq, *J* = 14.0, 1.5 Hz, 4H), 4.71 (s, 1H), 4.36 (dd, *J* = 5.8, 3.9 Hz, 2H), 4.30 (dd, *J* = 5.9, 3.9 Hz, 2H), 4.07 (tt, *J* = 7.2, 3.6 Hz, 2H), 3.26 (dd, *J* = 13.8, 7.1 Hz, 2H), 3.04 (dd, *J* = 13.8, 7.2 Hz, 2H), 2.32 (s, 3H), 1.16 (t, *J* = 7.1 Hz, 3H); ^13^C NMR (151 MHz, CDCl_3_): *δ* 165.73 (s), 158.18 (s), 153.75 (s), 146.22 (s), 136.73 (s), 133.68 (s), 127.89 (s), 123.87 (s), 123.85 (s), 117.75 (s), 116.24 (s), 116.11 (s), 115.33 (s), 114.76 (s), 101.48 (s), 68.16 (s), 66.46 (s), 59.99 (s), 55.05 (s), 49.45 (s), 35.31 (s), 18.56 (s), 14.21 (s). HRMS (ESI) m/z for C_29_H_33_FN_2_O_5_S_2_ [M+Na]^+^ calcd 595.1707, found 595.1710.

### 4.2 Cell Culture

RAW 264.7 mouse macrophage cell line (RAW264.7) were purchased from BNCC (BeNa Culture collection, China). RAW264.7 cell were cultured in DMEM containing 10% fetal bovine serum, 100 units/mL penicillin, and 100 µg/mL streptomycin at 37°C in a 5% CO_2_ humidified atmosphere. Cells in logarithmic growth phase are used for later experimental research.

### 4.3 Cell Cytotoxicity

RAW264.7 cells (5 *10^3^ cells/well) were seeded into 96‐well plate and cultured overnight. Compounds (6.25 µM, 12.5 µM, 25 µM, 50 µM) treated cell for 24 h. 20 µL of MTT solution (5 mg/mL, Sigma‐Aldrich) was then added and incubated for an additional 4 h. The cell culture supernatants were removed and DMSO (150 µL) was added to added into per well for dissolving the resulting crystals. The absorbance value (OD value, 492 nm) was recorded byabsorbance microplate reader (MQX200, Bio‐Tek, USA).

### 4.4 Assay for NO Production

In this study, RAW264.7 cells were cultured in 48‐well tissue culture plates at a density of 6‐7*10^4^ cells/mL and allowed to adhere for overnight. Cells were pre‐treated with the test compounds at four different concentrations (e.g., 0.08, 0.4, 2, 10 µM), or vehicle control for 1 h, followed by exposure to LPS (0.5 µg/mL). Control wells received only the vehicle with no LPS‐stimulation. Indomethacin was used as positive control. After incubation at 37°C for 24 h, the supernatants were collected and examined for NO production using Griess reagent (Beyotime Biotechnology, China) as previously described.

### 4.5 Western Blot

RAW264.7 cells in logarithmic growth phase were seeded at a density of 2‐3×10^6^ cells/well in a 6‐well plate and cultured overnight. Treat cells with different concentrations of compounds (0.5, 1.0, 2.0 µM) for 1 h, and stimulate with LPS (0.5 µg/mL) for 30 min or 24 h. Subsequently, cells were lysed using RIPA lysis buffer and protein concentration was measured using BCA protein assay kit (Beyotime Biotechnology, China). Separate proteins using SDS‐PAGE electrophoresis and transfer them onto PVDF membranes. Incubate in TBS/Tween‐20 containing 5% skim milk powder for 2 h, and incubate PVDF membrane with specific primary antibody overnight at 4°C. After washing three times, co‐incubate with HRP conjugated secondary antibody for 1 h and analyze using chemiluminescence.

### 4.6 Compound D3 Alleviates DSS‐Induced Acute Colitis in Mice

All experiments and animal care procedures were approved by the Animal Resource Center of Anhui Medical University by the National Institutes of Health Guide for the Care and Use of Laboratory Animals (20230875). Randomly selected healthy C57BL/6 mice were divided into the following five groups (10 mice per group): Normal group (0.5% carboxymethyl cellulose sodium), DSS model group (3% DSS), sulfasalazine control group (200 mg/kg), D3 low‐dose treatment group (3% DSS + 30 mg/kg), and **D3** high‐dose treatment group (3% DSS + 60 mg/kg). Following the aforementioned grouping, experimental mice in each group were administered 3% DSS dissolved in distilled water, while normal mice received distilled water alone. Daily oral gavage administration was conducted for the experimental groups, with normal groups receiving an equivalent volume of carboxymethyl cellulose sodium. Throughout the administration period, pathological symptoms such as body weight, stool consistency, and rectal bleeding were recorded daily. Disease Activity Index (DAI) scores were calculated based on recorded data, considering parameters including weight loss, stool consistency, and rectal bleeding. On day 10 post‐administration, mice were euthanized via cervical dislocation, followed immediately by laparotomy and collection of colon tissues from the anus to just above the cecum. Colon length was measured after flushing with pre‐chilled physiological saline, and tissues were fixed in 3% formaldehyde and subjected to histopathological analysis using HE staining. Inflammatory cell infiltration was scored as follows: 0, none; 1, mild infiltration; 2, moderate infiltration; 3, severe infiltration extending into deeper layers. Crypt damage was scored as follows: 0, intact crypts; 1, loss of the basal one‐third of crypts; 2, loss of the basal two‐thirds of crypts; 3, only surface epithelium remaining; 4, complete loss of crypts and epithelium.

### 4.7 Acute Toxicity Study

To assess the acute toxicity of compound **D3** in vivo, healthy C57BL/6 mice (15–18 g, half male and half female) were randomly divided into two groups (*n* = 5 per group). Compound **D3** was administered orally as a single dose of 1000 mg/kg, while control mice received an equal volume of normal saline. Animals were observed daily for 14 days after treatment. Clinical signs of toxicity, including changes in behavior, fur condition, food consumption, locomotor activity, lethargy, hyperactivity, tremors, convulsions, and mortality were recorded. Body weights were monitored throughout the experimental period. At the end of the observation period, mice were euthanized, and major organs, including the heart, liver, spleen, lungs, and kidneys were collected for histopathological examination following H&E staining to evaluate potential tissue toxicity.

### 4.8 Statistical Analyses

Data are presented as mean ± SD. Statistical analyses were performed using GraphPad Prism software. Comparisons among multiple groups were conducted using one‐way ANOVA followed by Tukey's multiple‐comparison post hoc test. A value of *p* < 0.05 was considered statistically significant. The value of n represents the number of independent animals used in each group.

## Author Contributions

**Rui Dong**, **Jiaying Chen**, and **Si‐Yu Li** performed the synthetic experiments and analyzed the data. **Jian‐wei Jiang** and **Huan Wang** performed the bioassays and analyzed the data. **Guo‐Ping Zhang** conceived and wrote the article.

## Conflicts of Interest

The authors declares no conflict of interest.

## Supporting information




^1^H NMR, ^13^C NMR (Figures S1–S60), and HR‐MS (Figures S60–S90) spectra data are provided as Supporting Information.
**Supporting File 1**: cbdv71729‐sup‐0001‐SuppMat.docx.

## Data Availability

The data that supports the findings of this study are available in thesupplementary material of this article.
