# Supplementary material for: Development of Dithioacetal‐Modified Dihydropyrimidone Derivatives with Potential Anti‐Inflammatory Activity for Inflammatory Bowel Disease
Source: Chem Biodivers. 2026 Sep 25;23(9):e71729. doi: 10.1002/cbdv.71729 (PMC13614288; doi:10.1002/cbdv.71729)
Supplement: Supplementary file 1 [file CBDV-23-e71729-s001.docx]

**Development of Dithioacetal-Modified Dihydropyrimidone Derivatives with Potential Anti-inflammatory Activity for Inflammatory Bowel Disease**

Jian-Wei Jiang^a^ , Rui Dong^a^, Qi Cao^a^, Huan Wang^a^, Si-Yu Li^a^, Guo-Ping Zhang*^,a,b^, Qian-Yi Wang*^,a,b^

*^a^Key Laboratory of Green and Precise Synthentic Chemistry and Applications, Ministry of Education, Huaibei Normal University, Huaibei 235000, P. R. China.*

*^b^College of Chemistry and Chemical Engineering, Huaibei Normal University, Huaibei 235000, P. R. China.*

Corresponding author: wangqy@chnu.edu.cn and hbzgp-1@163.com

**1.^1^H and ^13^C NMR Spectra of D1-D30………… ………………………… ….2-31**

**2.HR-MS Spectra of D1-D30………………………………………………….32-41**

**^1^H and ^13^C NMR Spectra of D1-D30**

**Supplementary Figure S1** ^1^HNMR spectra of compound **D1**

**^13^CNMR spectra of compound D1**

**Supplementary Figure S2 ^13^CNMR spectra of compound D1**

**Supplementary Figure S3** ^1^HNMR spectra of compound **D2**

**Supplementary FigureS 4** ^13^CNMR spectra of compound **D2**

**Supplementary Figure S5** ^1^HNMR spectra of compound **D3**

**Supplementary Figure S6** ^13^CNMR spectra of compound **D3**

**Supplementary Figure S7** ^1^HNMR spectra of compound **D4**

**Supplementary Figure S8** ^13^CNMR spectra of compound **D4**

**Supplementary Figure S9** ^1^HNMR spectra of compound **D5**

**Supplementary Figure S10** ^13^CNMR spectra of compound **D5**

**Supplementary Figure S11** ^1^HNMR spectra of compound **D6**

**Supplementary Figure S12** ^13^CNMR spectra of compound **D6**

**Supplementary Figure S13** ^1^HNMR spectra of compound **D7**

**Supplementary Figure S14** ^13^CNMR spectra of compound **D7**

**Supplementary Figure S15** ^1^HNMR spectra of compound **D8**

**Supplementary Figure S16** ^13^CNMR spectra of compound **D8**

**Supplementary Figure S17** ^1^HNMR spectra of compound **D9**

**Supplementary Figure S18** ^13^CNMR spectra of compound **D9**

**Supplementary Figure S19** ^1^HNMR spectra of compound **D10**

**Supplementary Figure S20** ^13^CNMR spectra of compound **D10**

**Supplementary Figure S21** ^1^HNMR spectra of compound **D11**

**Supplementary Figure S22** ^13^CNMR spectra of compound **D11**

**Supplementary Figure S23** ^1^HNMR spectra of compound **D12**

**Supplementary Figure S24** ^13^CNMR spectra of compound **D12**

**Supplementary Figure S25** ^1^HNMR spectra of compound **D13**

**Supplementary Figure S26** ^13^CNMR spectra of compound **D13**

**Supplementary Figure S27** ^1^HNMR spectra of compound **D14**

**Supplementary Figure S28** ^13^CNMR spectra of compound **D14**

**Supplementary Figure S29** ^1^HNMR spectra of compound **D15**

**Supplementary Figure S30** ^13^CNMR spectra of compound **D15**

**Supplementary Figure S31** ^1^HNMR spectra of compound **D16**

**Supplementary Figure S32** ^13^CNMR spectra of compound **D16**

**Supplementary Figure S33** ^1^HNMR spectra of compound **D17**

**Supplementary Figure S34** ^13^CNMR spectra of compound **D17**

**Supplementary Figure S35** ^1^HNMR spectra of compound **D18**

**Supplementary Figure S36** ^13^CNMR spectra of compound **D18**

**Supplementary Figure S37** ^1^HNMR spectra of compound **D19**

**Supplementary Figure S38** ^13^CNMR spectra of compound **D19**

**Supplementary Figure S39** ^1^HNMR spectra of compound **D20**

**Supplementary Figure S40** ^13^CNMR spectra of compound **D20**

**Supplementary Figure S41** ^1^HNMR spectra of compound **D21**

**Supplementary Figure S42** ^13^CNMR spectra of compound **D21**

**Supplementary Figure S43** ^1^HNMR spectra of compound **D22**

**Supplementary Figure S44** ^13^CNMR spectra of compound **D22**

**Supplementary Figure S45** ^1^HNMR spectra of compound **D23**

**Supplementary Figure S46** ^13^CNMR spectra of compound **D23**

**Supplementary Figure S47** ^1^HNMR spectra of compound **D24**

**Supplementary Figure S48** ^13^CNMR spectra of compound **D24**

**Supplementary Figure S49** ^1^HNMR spectra of compound **D25**

**Supplementary Figure S50** ^13^CNMR spectra of compound **D25**

**Supplementary Figure S51** ^1^HNMR spectra of compound **D26**

**Supplementary Figure S52** ^13^CNMR spectra of compound **D26**

**Supplementary Figure S53** ^1^HNMR spectra of compound **D27**

**Supplementary Figure S54** ^13^CNMR spectra of compound **D27**

**Supplementary Figure S55** ^1^HNMR spectra of compound **D28**

**Supplementary Figure S56** ^13^CNMR spectra of compound **D28**

**Supplementary Figure S57** ^1^HNMR spectra of compound **D29**

**Supplementary Figure S58** ^13^CNMR spectra of compound **D29**

**Supplementary Figure S59** ^1^HNMR spectra of compound **D30**

**Supplementary Figure S60** ^1^HNMR spectra of compound **D30**

**HR-MS Spectra of D1-D30**

**Supplementary Figure S61** HR-MS of Compound **D1**

**Supplementary Figure S62** HR-MS of Compound **D2**

**Supplementary Figure S63** HR-MS of Compound **D3**

**Supplementary Figure S64** HR-MS of Compound **D4**

 **Supplementary Figure S65** HR-MS of Compound **D5**

 **Supplementary Figure S66** HR-MS of Compound **D6**

 **Supplementary Figure S67** HR-MS of Compound **D7**

**Supplementary Figure S68** HR-MS of Compound **D8**

**Supplementary Figure S69** HR-MS of Compound **D9**

**Supplementary Figure S70** HR-MS of Compound **D10**

**Supplementary Figure S71** HR-MS of Compound **D11**

**Supplementary Figure S72** HR-MS of Compound **D12**

**Supplementary Figure S73** HR-MS of Compound **D13**

**Supplementary Figure S74** HR-MS of Compound **D14**

**Supplementary Figure S75** HR-MS of Compound **D15**

**Supplementary Figure S76** HR-MS of Compound **D16**

**Supplementary Figure S77** HR-MS of Compound **D17**

**Supplementary Figure S78** HR-MS of Compound **D18**

**Supplementary Figure S79** HR-MS of Compound **D19**

**Supplementary Figure S80** HR-MS of Compound **D20**

**Supplementary Figure S81** HR-MS of Compound **D21**

**Supplementary Figure S82** HR-MS of Compound **D22**

**Supplementary Figure S83** HR-MS of Compound **D23**

**Supplementary Figure S84** HR-MS of Compound **D24**

**Supplementary Figure S85** HR-MS of Compound **D25**

**Supplementary Figure S86** HR-MS of Compound **D26**

**Supplementary Figure S87** HR-MS of Compound **D27**

**Supplementary Figure S88** HR-MS of Compound **D28**

**Supplementary Figure S89** HR-MS of Compound **D29**

**Supplementary Figure S90** HR-MS of Compound **D30**
